# The CD226/TIGIT axis is involved in T cell hypo-responsiveness appearance in long-term kidney transplant recipients

**DOI:** 10.1038/s41598-022-15705-6

**Published:** 2022-07-12

**Authors:** Arnaud Del Bello, Anna Gouin, Camille Chaubet, Nassim Kamar, Emmanuel Treiner

**Affiliations:** 1 Nephrology and Organ Transplant Department, CHU Toulouse, 1 av Jean Poulhès, 31059 Toulouse Cedex 9, France; 2 Université Paul Sabatier Toulouse III, Toulouse, France; 3 Toulouse Institute for Infectious and Inflammatory Diseases (Infinity), INSERM UMR1043-CNRS 5282, Toulouse, France; 4 Laboratory of Immunology, Biology Department, CHU Toulouse, Toulouse, France; 5 Infinity—Inserm UMR 1291-CNRS UMR5051, CHU Purpan, BP3028, 31024 Toulouse Cedex 3, France

**Keywords:** Immunology, Nephrology

## Abstract

T cell exhaustion refers to a dysfunctional state in which effector T cells present a decreased ability to proliferate and to produce cytokines, while the co-expression of inhibitory receptors increases. We investigated global and donor-specific T cell responses in a cohort of stable, living-donor kidney transplant patients that received similar immunosuppression. After transplantation, an increase in the ratio of TIGIT + /CD226 + in mCD4 + T cells (r = 0.47, p = 0.01), and a decrease of CD226 + TIGIT-mCD4 + T cells was observed (r = − 0.55, p = 0.001). This leads to an increase of dysfunctional T cells in patients far from transplantation. In mCD8 + T cells, a decrease of IL-2 production after mitogenic stimulation was observed far from transplantation. Phenotypic analyses revealed an increase of mCD8 + T cells co-expressing PD-1 and TIGIT over time (r = 0.51, p = 0.02). After donor-specific stimulation, the ability of CD4 + T cells to proliferate was decreased compared with third parties. CD4 + T cells expressing CD226 and TIGIT were correlated with allospecific CD4 + proliferation (r = 0.68, *p* = 0.04). Our study suggests that after kidney transplantation a T cell hyporesponsiveness appears over time, driven by a dysregulation of CD226/TIGIT axis in mCD4 + T cells, associated with an increase of PD1 + TIGIT + in mCD8 + T cells.

## Introduction

Despite significant improvement**,** long-term kidney allograft survival rates remain disappointing^1,2^. Infectious and neoplastic-related complications represent major pitfalls after transplantation^3^, while the risk of rejection dictates the need to maintain immunosuppressive therapy^4^. Nonetheless, the phenotype of rejections evolves over time post-transplantation, with a decreased prevalence of acute T-cell mediated rejection, and an increase of chronic antibody-mediated rejection^5,6^. However, the immunological mechanisms underlying this phenomenon are being gradually unraveled^6^.

T cell exhaustion (Tex) refers to a dysfunctional state in which effector T cells present a decreased ability to proliferate, to secrete cytokines (e.g., interleukin [IL]-2, interferon [IFN]-γ, tumor or necrosis factor [TNF]-α), and co-express several inhibitory receptors (IRs) (e.g., programmed cell death protein 1 [PD-1], T cell immunoglobulin and mucin-domain containing-3 [Tim-3], 2B4, CD160, T cell immunoreceptor with Immunoglobulin and ITIM domains [TIGIT]) after chronic exposure to antigen stimulation^7,8^. High antigen load and prolonged antigen exposure are the main factors for the development of this dysfunctional state^8^. Tex was described in human persistent viral infection (mainly HCV^9^, HBV^10^, HIV^11^) and cancer^12–14^, leading to new revolutionizing therapies^15,16^. Differences in immunogenicity and microenvironment prevent the use of a universal signature of Tex^8^. Consequently, Tex must be studied in a situation-dependent manner. Among the different co-stimulatory and co-inhibitory receptor pathways, some of them were recently highlighted. For example, the dysregulation of the IR TIGIT and CD226 (a stimulatory receptor that shares CD112 and CD155 ligands with TIGIT) expression in T cells was recently suspected to be involved in severe forms of autoimmune diseases^17,18^, as well as in cancer and chronic viral infections^19–21^.

In solid organ transplantation, donor-reactive T cells from immunosuppressed recipients are chronically exposed to a high load of the transplanted organ, which is a prerequisite to the development of Tex. Some evidence of a positive association between graft outcomes and Tex after transplantation was previously highlighted in murine models^22,23^. However, little is known concerning the development of exhaustion after transplantation in humans. Fribourg and colleagues^24^ previously found that exhausted CD4 + and CD8 + T cells increased during the first months' post-transplantation, particularly in patients who received depleting induction. They also described a negative correlation between graft fibrosis and the percentage of exhausted cells. Our group previously investigated the expression of IRs and function of T cells in ABO and HLA-incompatible kidney transplant recipients and found that TIGIT was dramatically increased in HLA-incompatible recipients leading to alteration of T cell function^25^.

To date, the function and exhaustion-associated phenotype of T cells long after kidney transplantation have been poorly documented. In this study, we followed the progression of T cell phenotype and function with time in a cross-sectional cohort of stable living kidney transplant donors receiving standard immunosuppressive therapy, and uncovered the phenotype associated with the development of hyporesponsiveness in CD4 and CD8 T cells.

## Patients and methods

### Patient selection

#### Kidney transplant recipients

From 2008 to 2019, 357 first transplant, non-combined, living-donor kidney transplantations were performed in the Nephrology and Organ Transplant Department of CHU de Toulouse, France. Excluded were all patients with a history of graft rejection or reoccurrence of initial kidney disease, Donor-Specific Antibodies (preformed or de novo), post-transplant CMV or BK-virus viremia, patients that had received induction therapy with a B or T-cell depleting agent, as well as patients that had not received any immunosuppressive treatment with tacrolimus, mycophenolic acid, and steroids (n = 304). Hence, 53 patients were included in the study. Among them, peripheral blood mononuclear cells (PBMCs) from the kidney donor were available for eight recipients.

#### Dialysis patients

Twenty-one patients on the waitlist for kidney transplantation but who had not received any immunosuppressive treatments were also analyzed.

All patients included in the study gave their informed consent (Nephrogen cohort (DC-2011-1388). All methods were carried out in accordance with relevant guidelines and regulations**.**

This study was approved by Toulouse University Hospital and confirm that ethic requirements were totally respected.

### Isolation of PBMCs

PBMCs were isolated from patients’ blood samples by centrifugation in density gradient medium (Ficoll-Paque). After washing, PBMCs were frozen in Dimethyl Sulfoxide (DMSO) at − 196 °C in liquid nitrogen and stored in the Centre de Ressources Biologiques (CRB) of the CHU of Toulouse, until further use.

#### Preparation of “third-party donors”

PBMC were isolated from buffy coats from four different healthy donors (obtained from the Etablissement Français du Sang (EFS). PBMC were mixed in equal numbers, and the pool of cells was aliquoted and frozen in liquid nitrogen until use.

### Phenotypic analyses

PBMC were thawed, washed and counted. Cell surface staining included an incubation with brilliant stain buffer (BD Biosciences), followed by a 30 min incubation at room temperature with the monoclonal antibodies at the appropriate concentration. For intracellular staining, cells were fixed and permeabilized with the transcription factor buffer set (BD Biosciences), followed by a 1-h incubation at 4 °C with the monoclonal antibodies at the appropriate concentration. After washing, cells were analyzed by flow cytometry. All patients were tested for exhaustion markers; when blood samples were available, T helper subsets, regulatory T cells, and differentiation panels were analyzed.

### T cell functional analysis

#### Polyclonal stimulation

PBMC were thawed, washed, counted, and plated at a concentration of 10^7^/ml in 96 well plates. Cells were then incubated in the absence or presence of 1 μg/ml phorbol 12-myristate 13-acetate and 2 μg/ml Ionomycin (both from Sigma-Aldrich) for 4 h, in the presence of Golgi plug (BD Biosciences). At the end of the incubation time, cells were harvested, and membrane staining was performed followed by fixation and permeabilization with the Fix/Perm kit as per the manufacturer’s instructions (BD Biosciences). Intracellular staining was performed at 4 °C with 1 h incubation with anti-IFNγ, TNFα, and IL-2 antibodies, followed by flow cytometry analysis.

#### Mixed lymphocyte reaction

Frozen PBMCs from graft donors or from the pool of healthy donors were thawed, washed, and resuspended in RPMI (ThermoFisher Medium). Cells were irradiated at 3000 rad, followed by a 45 min incubation at 37 °C with CMFDA (Thermofisher) in AIM V medium (Thermofisher). After washes, cells were suspended in AIM V medium at 10^7^/ml before use as stimulator cells.

Responder cells were prepared as follows: frozen PBMCs from transplant recipients were thawed, washed, and resuspended in RPMI-10% with FCS (ThermoFisher Medium). After counting, responding cells were incubated for 10 min in pre-warmed PBS with Cell Trace Violet (Thermofisher), washed with pure SVF, and then resuspended in AIMV medium at 10^7^/ml.

For the MLR, 250, 000 CTV-labeled responder cells and 250, 000 CMFDA-labeled, irradiated stimulators cells (donor-specific or third-party) were plated in a 96-well plate, and kept in an incubator at 37 °C 5% CO_2_ for 7 days. In some selected cases, these cultures were performed in duplicates.

At the end of the MLR, one of the duplicates was used for phenotypic analysis as already described. Cells were harvested from the second duplicate, washed, and stimulated with PMA and ionomycin in the presence of Golgi Plug as already described. After 4 h, cells were stained for cell surface markers, followed by fixation/permeabilization, and intracellular cytokine stainings with anti-IFNγ and anti IL-2 antibodies. Stained cells were analyzed by flow cytometry.

### Flow cytometry

Antibodies used for the different phenotypes and functional panel are listed in Supporting document 1.

Acquisitions were performed either on a Fortessa X18, a Fortessa X20 or a LSRII flow cytometer (BD Biosciences, San Jose, CA, USA). Data were analyzed with FlowJo software (v10.7, BD Biosciences).

### Clustering analyses

We first clustered cells using the Phenograph algorithm^26^, and t-SNE^27^ to visualize high-dimensional data in two dimensions while preserving single-cell resolution. To minimize variability in measurement, our analysis strategy was structured as follows:

- Equal contribution of the samples: to avoid bias in Phenograph for the subsets present in the samples, we first extracted and analyzed separately memory CD4 + and CD8 + T cells and maintained an equal contribution in the number of cells from every sample determined by the sample with the lowest number of CD4 + or CD8 + T cells.
- Reiteration: to increase the power of the analyses for the cell subsets with a low number of events, we reiterated the entirety of the sampling process and Phenograph, clustering up to 5 times to achieve robustness in the results.

### Statistical analysis

All statistical analyses were performed with Prism Software v8.1 (GraphPad, San Diego, CA, USA). Statistical tests used are reported in the figure legend. Unpaired parametric or non-parametric tests were chosen according to the Gaussian analysis of data. Spearman’s coefficient was used for correlation analyses. A *P*-value < 0.05 was considered statistically significant.

## Results

### Decreased polyclonal T cell responses in long-term kidney transplanted recipients

To investigate the possibility that kidney transplantation may induce a state of lymphocyte exhaustion, we undertook a cross-sectional study including 30 living-donor KTR studied at different times post-transplantation (Table 1). All patients were grafted with HLA-mismatched organs, and were under the same immunosuppressive regimen (excluding T cell-depleting agents). We also purposely selected only patients under stable conditions from the time of transplantation, i.e. without any history of cancer, active infection, CMV or BK virus replication, reoccurrence of the initial kidney disease, presence of DSA, or transplant rejection.

**Table 1:** Main characteristics of the 30 living-donor kidney transplant recipients included in the inhibitory receptors expression changes after transplantation.

Variable	Results
Recipient age (years), mean (± range)	52 ± 10
Recipient gender, male (%)	18 (60)
Donor age (years), mean (± range)	53 ± 13
Time between Tx-sample analysis (months), median (IQR 25–75)	39 (27; 71)
CKD-Epi estimated GFR at sample analysis, mL/min/1.73m2, mean (± range)	58 ± 21
Positive CMV serology, yes (%)	16 (53)
Positive EBV serology, yes (%)	30 (100)
Positive Toxoplasma Gondii serology, yes (%)	25 (83)
**Initial kidney disease: n **(**%**)	
Glomerular	14 (47)
PKD	11 (37)
Vascular	1 (3)
Unknown	4 (13)
**Donor-recipient HLA mismatches, mean **(**± range**)	
A, B mismatches	3.7 ± 2.4
DR, DQ mismatches	1.9 ± 1.3
A, B, DR, DQ mismatches	1.8 ± 1.4
**Anti-HLA sensitization**	
Anti-class I	4 (13)
Anti-class II	2 (7)
Anti-class I and II	1 (3)
Donor-Specific Antibodies	0
**Immunosuppressive therapy**	
Anti-CD25 at induction, yes (%)	12 (40)

*Tx* Transplantation, *GFR* Glomerular Filtration Rate.

**Figure 1:**
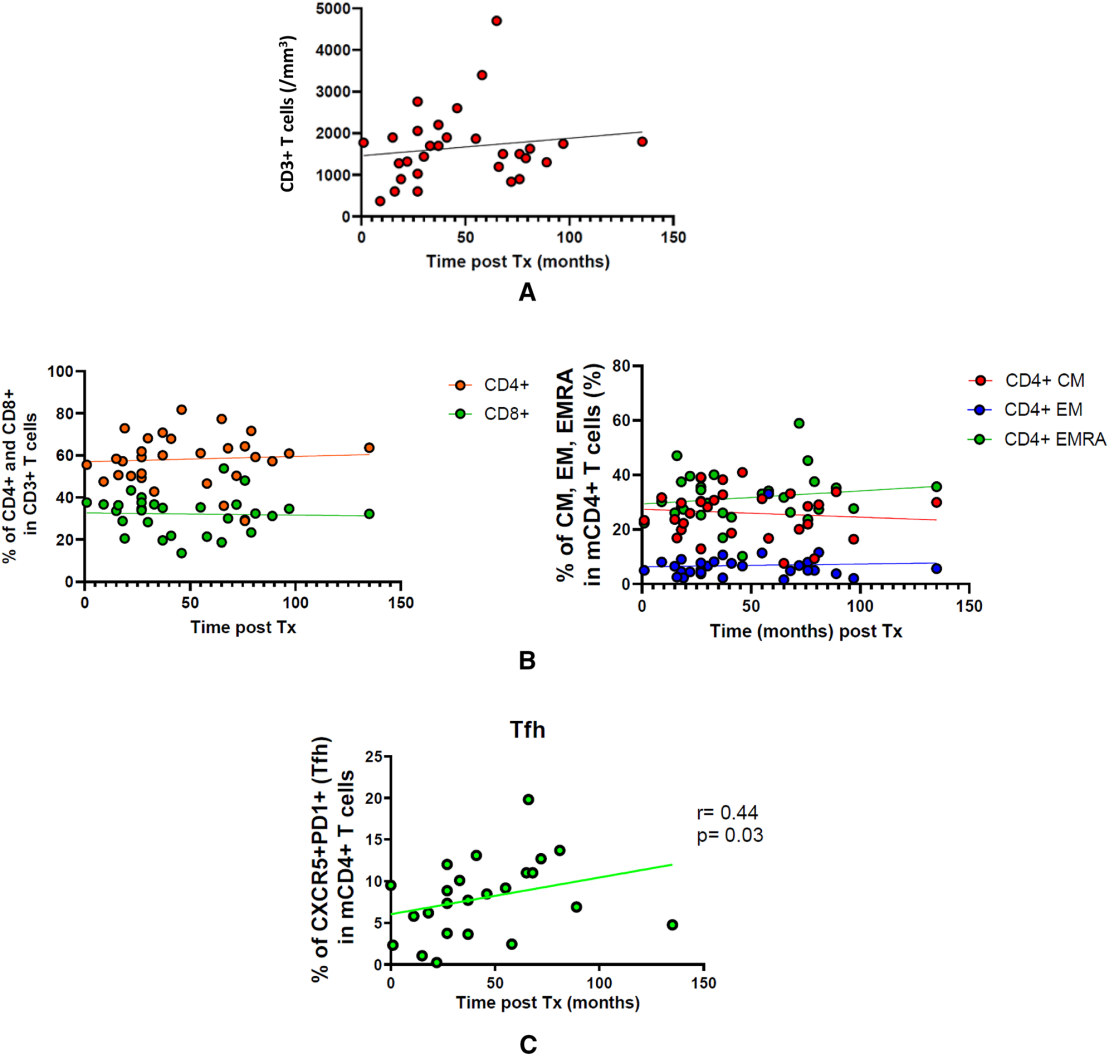
(**A–C**) Phenotype analyses of mCD4 + T cells. (**A**) CD3 + T cell number after transplantation. (**B**) CD4 + proportion among CD3 + (left) and memory T cell subsets of CD4 + (right). (**C**) Circulating T follicular helpers (due to blood sample availability, only 24 patients were tested).

We first analyzed the main circulating T lymphocyte subsets in patients at the time of study. The number of total CD3 + T cells, as well as CD4 + and CD8 + T cells, did not significantly vary with time from transplantation (Fig. 1A,B). Among CD4 + T cells, the proportion of memory cells remained steady (Fig. 1B and supporting Fig. 2A). Similarly, we analyzed the frequencies of CD4 + Tregs (Foxp3 + CD25hiCD127lo), of the various Thelper subsets (Th1, Th2, Th17 and Th17*, based on the relative expression of CCR6/CXCR3/CCR4), and of senescent/terminally differentiated CD28-CD57 + and KLRG1 + cells (supporting Fig. 2B,C). Although some of these subsets showed a dramatic inter-individual variation, they did not correlate with the delay post-transplantation (supporting Fig. 2C,D). By contrast, the subset of follicular helper T cells (CD45RA-CCR7-CXCR5 + PD1 +) significantly increased with time post transplantation (Fig. 1C). Altogether, most T cell subsets varied independently of the duration of contact between the graft antigens and the immune system of our patients.

**Figure 2:**
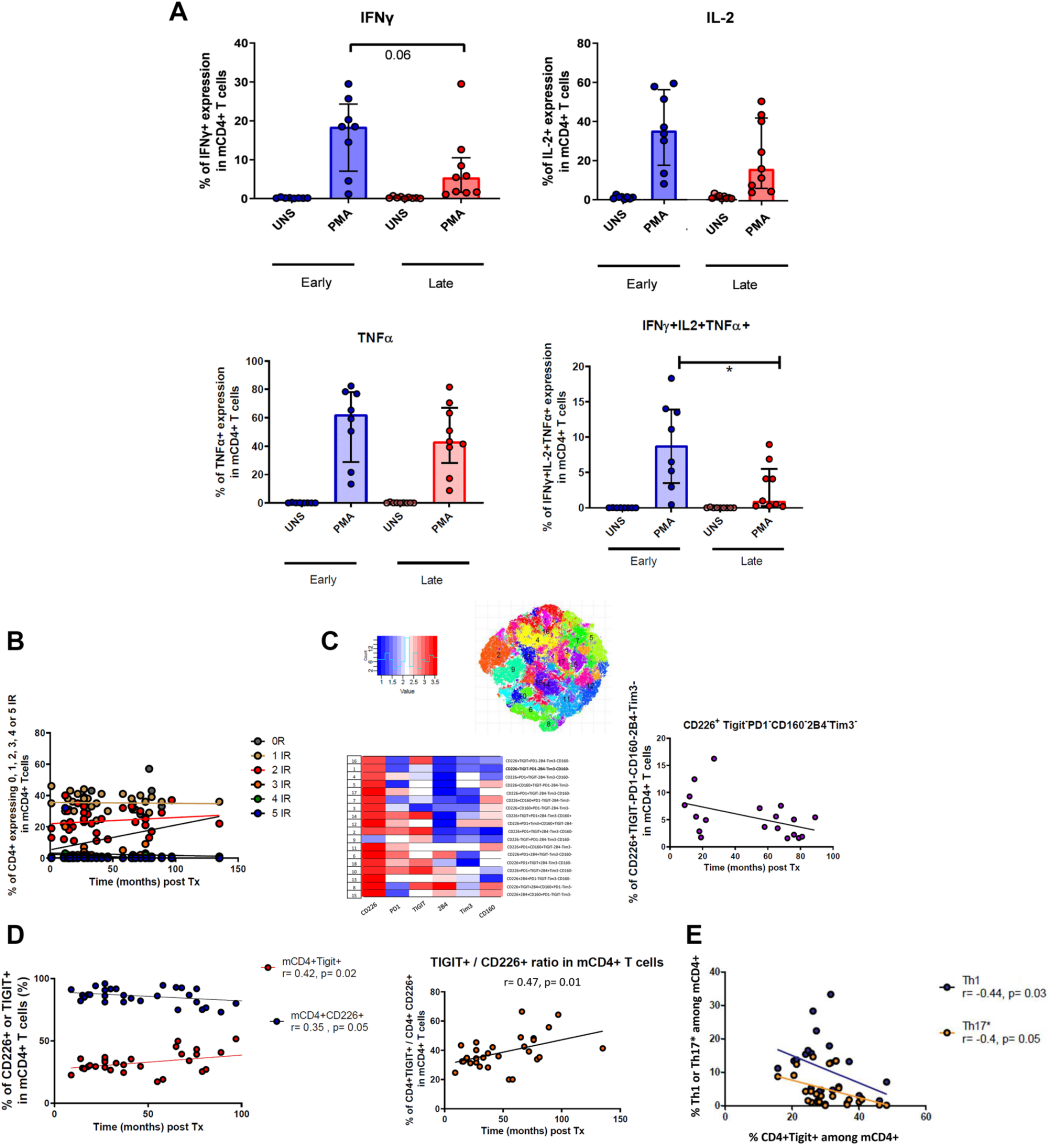
(**A–E**) Functional and phenotypic analyses of mCD4 + T cells. Comparisons were performed by an unpaired Mann–Whitney t-test. Statistical correlation was performed using a Spearman correlation test. (**A**) IFNγ, IL-2 and TNFα, and IFNγ + IL-2 + TNFα + production in mCD4 + T cells in unstimulated (UNS) or after PMA-ionomycin stimulation in early (< 3 months) and late (> 2 years) transplant patients. (**B**) Percentage of memory CD4 + T cells expressing 0, 1, 2, 3, 4, or 5 inhibitory receptors. (**C**) Unbiased clustering analysis of inhibitory receptor expression and CD226 expression in mCD4 + T cells after transplantation. (**D**) TIGIT, TIGIT + CD226 + expression in mCD4 + T cells (left) and TIGIT/CD226 expression ratio in memory CD4 + T cells. (**E**) Correlation between percentage of CXCR3 + CCR6- (Th1), and CXCR3 + CCR6 + (Th17*) subsets and percentage of TIGIT + CD4 + in memory CD4 + T cells.

Next, we measured the capacity of the patients CD4 + T cells to produce intracellular cytokines upon mitogenic stimulation with PMA and Ionomycin. We separately analyzed the response of T cells from recently (< 3 months) or late (> 2 years) transplanted patients (Table 2). As shown in Fig. 2A, CD4 + T cells from late KTR showed a dampened IFNγ response compared with early KTR, that also translated into a dramatic drop of triple (IFNγ, TNFα, IL-2) cytokine producers.

**Table 2:** Main characteristics of the 15 living-donor kidney transplant recipients included in the functional analysis.

Variable	Early (n = 7)	Late (n = 8)	Results
Recipient age (years), mean (± range)	51 ± 13	52 ± 14	0.73
Recipient gender, male (%)	5 (71)	6 (75)	0.99
Donor age (years), (± range)	55 ± 19	46 ± 14	0.48
**Donor-Recipient mismatches, mean (± range)**			
A, B mismatches	2.3 ± 0.5	3.0 ± 1.2	0.16
DR, DQ mismatches	1.9 ± 1.1	2.9 ± 0.9	0.11
A, B, DR, DQ mismatches	4.1 ± 0.9	5.9 ± 1.7	0.09
**Initial kidney disease**			0.45
Glomerular	4	2	
PKDA/genetic	2	4	
Unknown	1	2	
Time between Tx-sample analysis (months) median (IQR 25–75)	1 (1; 1)	64 (50; 83)	0.001
CKD-Epi eGFR at sample analysis, mL/min/1.73m2, mean (± range)	49 ± 17	70 ± 18	0.08
Positive CMV serology, yes (%)	5 (71)	4 (50)	0.61
Positive EBV serology, yes (%)	6 (86)	8 (100)	0.47

eGFR, estimated Glomerular Filtration Rate.

Exhausted T cells usually express simultaneously multiple surface receptors involved in the regulation of immune responses, referred to as inhibitory receptors (IR). An increase in the frequency of cells CD4 + exhausted T cells increase after kidney transplantation co-expressing multiple IR could possibly explain the observed blunted response of CD4 + T cells. We chose to focus on five important IR: PD1, TIGIT, 2B4, CD160 and Tim3, as well as CD226, a co-stimulatory receptor binding to the same ligands than TIGIT. The frequency of cells expressing any combination of 2 to 5 IR remained stable with time post-transplantation (Fig. 2B). Nevertheless, unbiased clustering analysis unraveled a time-dependent decrease in the frequency of cells expressing CD226 but none of the 5 IR included in the analysis (Spearman r = − 0.52, p = 0.02), (Fig. 2C). In other words, the frequency of cells expressing no IR decreases with time, which implies that expression of at least one of these IR must increase in a time-dependent fashion. Thus, we looked at the expression of specific IR. The expression of the 5 IR analyzed was similar between the whole cohort of Tx patients and dialysis controls (supporting Fig. 2E). However, we observed a time-dependent increase in the frequency of cells expressing TIGIT (T cell Immunoreceptor with Ig and ITIM domains) (Fig. 2D). TIGIT display multiple mechanisms to inhibit T cell responses. One of these involves the stimulatory receptor CD226, which is specific for the same ligands than TIGIT, but with lower affinity. CD226 expression tended to decrease with time post-transplantation (Fig. 2D). We observed that the increase in TIGIT expression with time involved both TIGIT + CD226- and TIGIT + CD226 + subsets, resulting in an increase in the ratio of TIGIT + /CD226 + cells among the CD4 + subset (Fig. 2D). Importantly, we found a negative correlation between TIGIT + cells and Th1 (Spearman r = − 0.44, p = 0.03) or Th17* (r = − 0.40, p = 0.05) subsets (both involved in T cell-mediated rejection), strongly suggesting that the expansion of TIGIT + cells dampens T cell-mediated Immunity in vivo (Fig. 2E).

**Figure 3:**
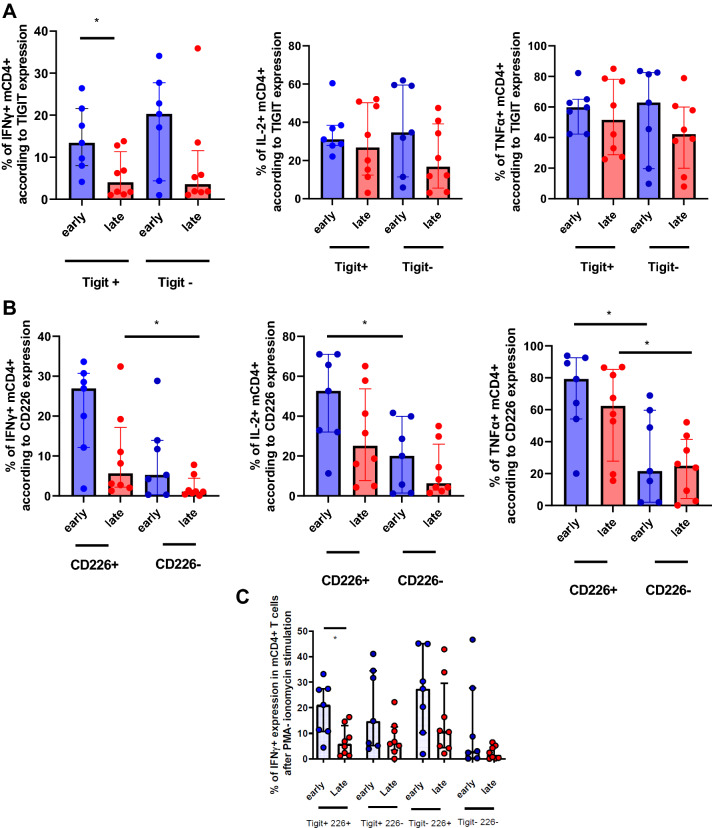
(**A**–**C**) Functional analyses according to TIGIT and CD226 expression in mCD4 + T cells in early (< 3 months) and late (> 2 years) kidney transplant patients. Comparisons were performed by an unpaired Mann-Whitney t-test (*p < 0.05, ** p < 0.01, ***p < 0.001). (**A**) Comparison of IFNγ, IL2 and TNFα production in mCD4 + T cells according to the expression of TIGIT in patients recently transplanted and those far from transplantation. (**B**) Comparison of IFNγ, IL2 and TNFα production in mCD4 + T cells according to the expression of CD226 in patients recently transplanted and those far from transplantation. (**C**) Comparison of IFNγ production in mCD4 + T cells according to the expression of TIGIT and CD226 in patients recently transplanted and those far from transplantation.

We next asked whether TIGIT and/or CD226 expression was directly responsible for T-cell mediated hyporesponsiveness in late KTR. Intracellular production of IFNγ, IL-2 or TNFα after mitogenic stimulation was similar between TIGIT + and TIGIT-CD4 + T cells (Fig. 3A). However, IFNγ production by TIGIT + mCD4 + T cells was higher in early KTR comparing with late KTR (Fig. 3A). On the other hand, CD226 expression was associated with cytokine production (Fig. 3B). This was particularly observed in early KTR (Fig. 3B). When we analyzed the production of cytokines by CD226/TIGIT-defined subsets according to time post-transplantation, we found a significant drop in IFNγ production only in the TIGIT + CD226 + subset in late KTR (Fig. 3C). Altogether, our results strongly suggests that both TIGIT and CD226 expression regulates cytokine production in KTR, and that a dysregulation of this TIGIT/CD226 axis may be responsible for CD4 + hyporesponsiveness in late KTR.

### CD8 + exhausted T cells increase after kidney transplantation

**Figure 4:**
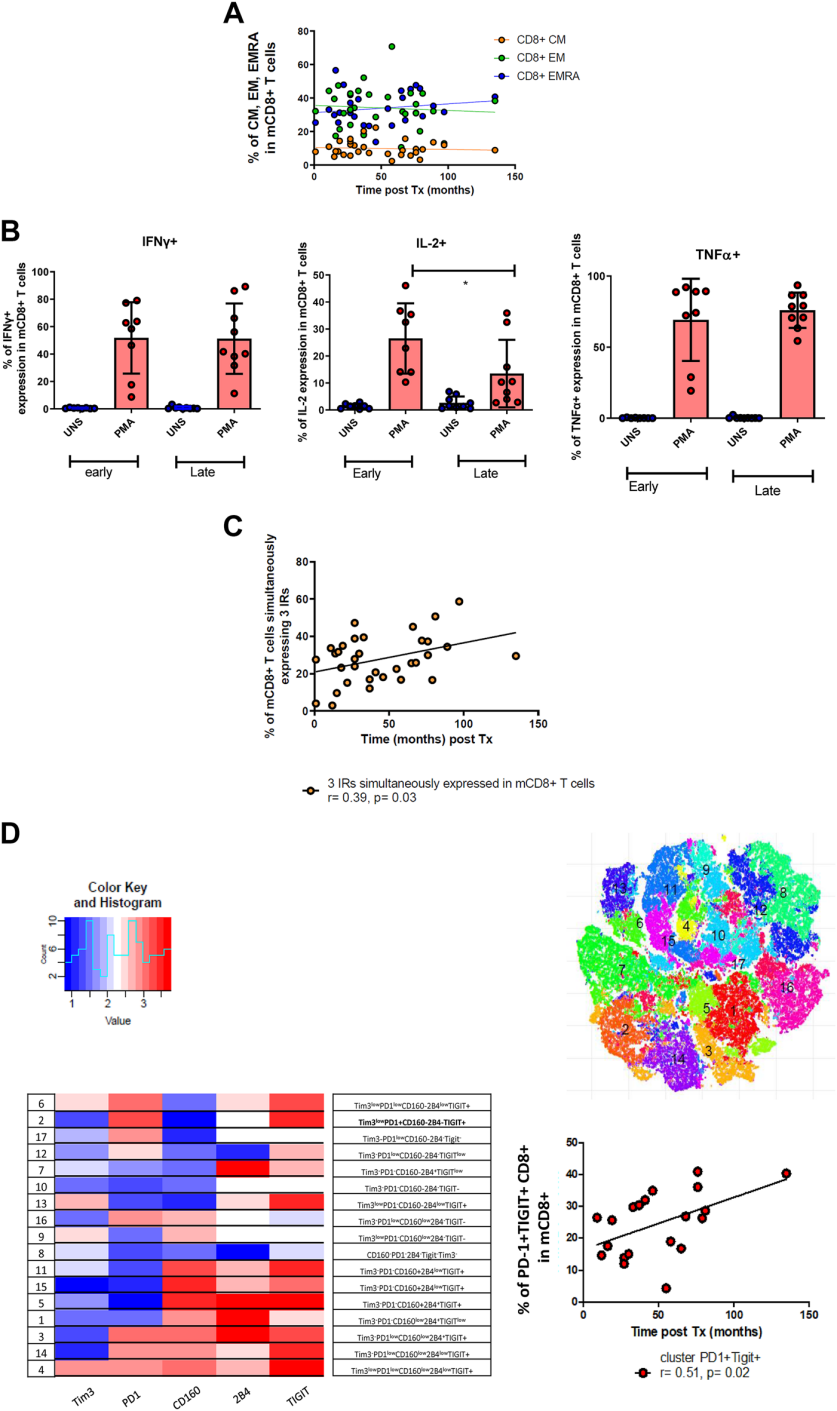
(**A**–**D**). Phenotype and functional analyses of mCD8 + T cells. Comparisons were performed by an unpaired Mann-Whitney t-test. Statistical correlation was performed using a Spearman correlation test. (*p < 0.05). (**A**) Memory CD8 + subsets over time post-transplantation. (**B**) Comparison of IFNγ, IL2, and TNFα production in mCD8 + in the recently transplanted and those far from transplantation. (**C**) Percentage of mCD8 + T cells that express 3 inhibitory receptors over time post-transplantation. (**D**) Unbiased clustering analysis of the different inhibitory receptors.

We next turned to the CD8 + T cell compartment within the same cohort (Tables 1 and 2). The memory CD8 + T cell subsets, as well as the different Tc1, Tc2, Tc17, and Tc17* subsets or CD57, CD28, KLRG1 expression were stable over time (Fig. 4A, supporting Fig. 2F,G). Upon mitogenic stimulation, CD8 + T cells from late KTR produced significantly less IL-2 than recently transplanted patients (Fig. 4B). The expression of each individual IR did not differ with time post-transplantation or compared with patients under dialysis (Supporting Fig. 2H). However, the percentage of CD8 + T cells simultaneously expressing three IRs increased with time post-transplantation (Fig. 4C). After unbiased clustering strategy, we observed that PD1 + TIGIT + mCD8 + T cells increase over time post-transplantation (Spearman r = 0.51, p = 0.02), (Fig. 4D).

Thus, akin to CD4 + T cells, we observed that CD8 + T cell hypo-responsiveness develop in long-term KTR, and is associated with the expansion of an exhausted PD1 + TIGIT + subset.

### Alloreactive CD4 + donor-specific hyporesponsiveness after kidney transplantation is correlated with CD4 + CD226 + TIGIT + Tim3-PD1^low^2B4^−^ T cells

We wanted to investigate whether an antigen-specific phenomenon directly linked to chronic exposure to graft alloantigens, could be uncovered. Thus, we selected 11 stable patients grafted from living donors at different time post-transplantation (Table 3), and analyzed their T cell reactivity by Mixed Lymphocyte Reaction (MLR) against donor cells (donor-specific alloreactivity) and against third-party cells (non-specific alloreactivity) (supporting document 3A, 3B).

**Table 3:** Main characteristics of the 11 patients included for the donor-specific response analyses.

Variable	Result
Recipient age at Tx (median), IQR (25–75)	42 ± 17
Recipient sex, male yes (%)	3 (38)
Recipient CMV serology positive (%)	4 (50)
Recipient EBV serology positive (%)	7 (88)
Time between Tx-blood sample analysis (months), IQR (25–75) *	3 (3; 11)
Anti-class I/II anti-HLA sensitization, yes (%)	2 (25)
Donor-Specific Antibodies at blood sample analysis, yes	0
Anti-CD25 induction therapy, yes (%)	4 (50)

*One patient provided blood samples for investigation at 0.5, 1-, and 3-months post-transplantation, and one other patient provided blood samples for investigation at 3- and 6-months post-transplantation.

**Figure 5:**
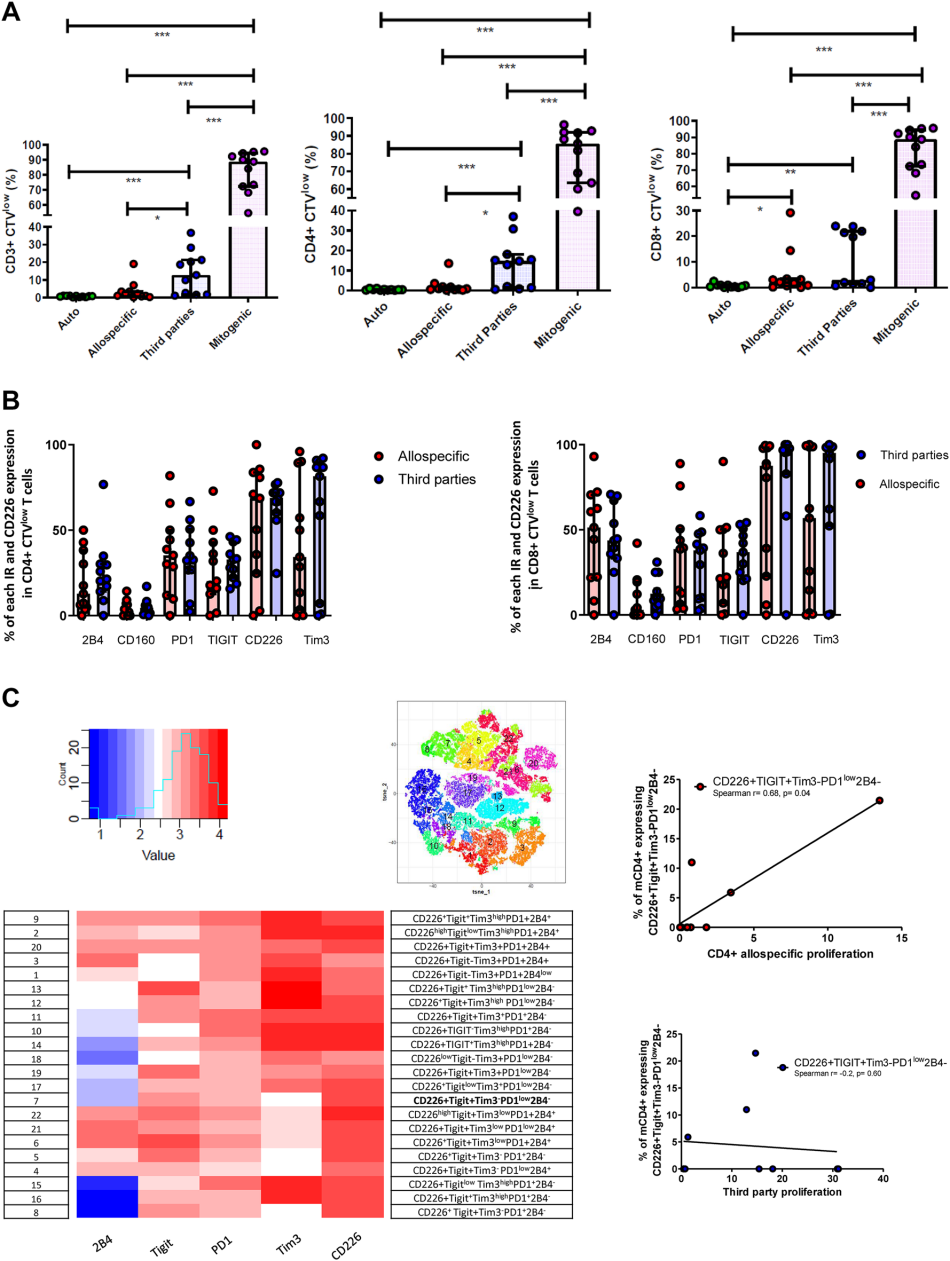
(**A–C**) Donor -specific and third-parties responses. (**A**) Proliferation of CD3 + , CD4 + and CD8 + T cells after autologous, allospecific, third-parties, and mitogenic stimulation. Comparisons were performed by an unpaired Mann–Whitney t-test. *: p < 0.05, ** p < 0.01, *** p < 0.0001. (**B**) Expression of each Inhibitory receptor in CTV^low^ CD4 + (left) and CD8 + (right) proliferating cells. (**C**) Unbiased clustering analysis of T cell phenotype before MLR, and correlation with donor-specific and third -party stimulation.

As shown in Fig. 5A, T cell proliferation measured at day 7 was lower after donor-specific stimulation compared with third-party (non-donor specific). This hypo-responsiveness was observed in CD4 + T cells only, but not for CD8 + T cells (donor-specific/third-party proliferation ratio (median [IQR]): 0.24 [0.04; 1.02], 0.06 [0.02; 0.92], 0.71 [0.03; 2.07] for CD3 + , CD4 + , CD8 + respectively). At the end of the MLR, PBMC were restimulated with PMA and Ionomycin for 4 h and analyzed for intracellular cytokine production (IFNγ, IL-2). No significant difference was observed with respect to cytokine production by CTV^low^ proliferating CD4 + and CD8 + T cells when comparing donor-specific or third party-stimulation (supporting Fig. 3C,D). Further, in either condition, intracellular cytokine production did not correlate with time post-transplantation (supporting Fig. 3C,D). We also analyzed the expression of IR and CD226 by proliferating, i.e. CTV^low^, T cells after MLR. As shown in Fig. 5B, a number of those receptors, mostly dominated by PD-1, CD226, TIGIT, and Tim3 are strongly expressed by proliferating cells However, there was strong inter-individual differences, but that did not correlate with time post-transplantation (Supporting Fig. 3E,F). Further, we found no significant differences between donor-specific and third-party MLR.

On the basis of an unbiased clustering analysis of recipient CD4 + and CD8 + exhaustion phenotype before MLR, the proportion of CD226 + TIGIT + Tim3-PD1^low^2B4^−^ CD4 + cells was correlated with allospecific, but not third-party, CD4 + proliferation (Spearman r = 0.68, p = 0.04). This observation was mainly driven by two recently transplant patients with high proportion of proliferating cells after MLR (Fig. 5C).

Hence, we observed an allospecific T cell hyporesponsiveness after kidney transplantation, mainly in CD4 + T cells. Donor-specific CD4 + T cell proliferation was correlated with the percentage of CD226 + TIGIT + Tim3-PD1^low^2B4^−^ CD4 + T cells, suggesting a decisive role of the CD226/TIGIT axis in donor-specific response.

## Discussion

In this study, we found that donor-specific T cell hyporesponsiveness prevails in CD4 + T cells after kidney transplantation and was linked with CD226 + TIGIT + CD4 + T cells. We also pointed out that polyclonal CD4 + T cells from patients far from transplantation presented a reduced IFNγ response after mitogenic stimulation which suggest a state of T cell exhaustion. Moreover, TIGIT expression increased in mCD4 + T cells after transplantation in stable conditions, at the expense of CD226 + TIGIT-mCD4 + T cells. As a result, we observed an altered function of this CD4 + T cell subset in recipients far from transplantation. The TIGIT/CD226 axis is a recently described pathway that regulates T cell function. Similar to other immune checkpoint pathways, such as CD28/CTLA4, CD226 presents a co-stimulatory function, that shares ligands with TIGIT which is a co-inhibitory receptor. Both of these receptors bind to two nectin and nectin-like proteins, CD155 (PVR) and CD112 (PVRL2). TIGIT is expressed on conventional αβ T cells, but also memory, regulatory, and follicular helper T cells^28^. TIGIT exerts an inhibitory function in several ways. TIGIT could regulate T cell responses via engagement in a cell-extrinsic manner via the ligation after homodimerization to CD155 in dendritic cells and macrophages (resulting in a shift of IL-12 production to IL-10), promoting a tolerogenic phenotype^29,30^. TIGIT could also exert an inhibitory function in a cell-intrinsic manner, by competing with CD226 for the binding of CD155, and interacting with CD226 on the surface of T cells to disrupt CD226 homodimerization^31^. In conventional CD4 + T cells, TIGIT expression exerts a direct inhibitory effect on T cell proliferation with inhibition of cytokine production, predominantly on IFNγ^32^. We also observed a negative correlation in kidney transplant recipients between the expression of TIGIT in conventional T cells, and the proportion of Th1 or Th17 subsets. Th1 and Th17 cells play a key role in the initiation and development of T cell rejection^33–35^. Taken together, our data suggest that in triple, CNI-based immunosuppressive therapy, increased TIGIT expression could participate in CD4 + T cell hyporesponsiveness and the previously described disappearance of T cell rejection over time^6^. Conversely, TIGIT expression on tumor-Infiltrating Lymphocytes of melanoma patients or on peripheral blood T cells of gastric cancer patients was previously associated with poor outcomes^36–38^. A decrease in TIGIT + CD4 + T cells was associated with severe forms of autoimmune diseases such as multiple sclerosis, atopic dermatitis, ulcerative colitis, and systemic lupus^39–42^. Moreover, dysregulation of CD226 + TIGIT + CD4 + T cell functions was previously described in severe forms of dermatomyositis^17^ and primary Sjögren syndrome^18^. CD226 downregulation and high levels of TIGIT were previously described in cancer and chronic viral infections^19–21^. In the same way, we previously found in highly immunosuppressed HLA incompatible kidney transplant recipients known to present a high risk of infectious complications^43^, higher levels of TIGIT expression, compared to ABO-incompatible recipients^25^. TIGIT was also previously identified in kidney biopsies as one of the most specific transcript changes (with other costimulation -related transcript) during pure T-cell mediated rejection, compared with other disease^44^. Finally, we observed that donor-specific T cell (but not third-parties) response involved preferentially CD226 + TIGIT + cells. The dysfunctional state observed in these cells far from transplantation could contribute to the donor-specific hyporesponsiveness observed after transplantation. Thus, exploring the TIGIT/CD226 after kidney transplantation could be a promising tool for monitoring the balance of immunosuppression after transplantation.

Conversely, we did not observe any increase of other inhibitory receptors such as PD-1, or any combination of inhibitory receptors (using clustering analyses), over time post-transplantation. In a mouse model of chronic rejection after heart transplantation, Sarraj and colleagues previously demonstrated an association between graft survival and CD4 + T cell exhaustion phenotype in the periphery, while Zou and colleagues^23^ demonstrated in a skin transplant model that donor antigen abundance is a main factor of T cell exhaustion development. In humans, Fribourg and colleagues previously found during the first 6 months post-transplantation, an increase of exhausted CD4 + T cell subsets, mainly represented by PD1 + TIGIT + 2B4-Tim3-cells. They also observed a negative correlation between CD4 + Tex and the development of graft fibrosis. Unlike the Fribourg study, we only included recipients that had not received T cell depleting agents, in order to avoid biases related to the expression of some inhibitory receptors during T cell reconstitution. Moreover, in our study, patients were far from transplantation and exposed to long-term use of immunosuppressive treatments. It was previously suggested in mouse models that T cell exhaustion could be prevented by calcineurin inhibitors^45,46^. Hence, it could be argued that CNI-based immunosuppression after transplantation could interfere with the development of exhaustion-induced hyporesponsiveness.

We also observed an increase of PD1 + TIGIT + CD8 + T cells after kidney transplantation. In cancer, TIGIT + PD1 + CD8 + cells were previously found to be upregulated and to present altered function^47,48^. Our results are consistent with those of Fribourg^24^ In their study, they found an increase of CD8 + Tex clusters mainly represented by PD1 + TIGIT + 2B4 + cells. They also found a correlation between graft fibrosis during the first months post-transplantation and the proportion of CD8 + Tex. Given these observations, monitoring the PD1 + TIGIT + CD8 + after transplantation could thus be an interesting path of investigation toward individualizing immunosuppression in the treatment of infections and cancer, or graft rejection and fibrosis.

Our study presents several limitations. First, we analyzed a relatively small sample of recipients. This was mainly driven by the ambition to homogenize patients: all transplant recipients included here received the same immunosuppressive treatment, did not present viral replication in blood, graft rejection, or reoccurrence of kidney disease. Moreover, despite the low number of patients, we were able to highlight some major changes in CD4 + and CD8 + T cell compartments after transplantation. Second, we were not able to investigate all previously described inhibitory receptors. We decided to focus on the main inhibitory receptors, associated with exhaustion development. Consequently, we cannot eliminate different signatures, with other IR after transplantation. For example, TOX was recently described as a major transcription factor for exhaustion in the mouse^49^ and should be investigated in future prospective studies. Nonetheless, using only the principal 5 IRs we described a dysregulation of the CD226/TIGIT axis and an increase of PD1 + TIGIT + CD8 + T cells. Conversely, a main strength of our study was the investigation of donor-specific T cell response according to exhaustion. Nonetheless, since exhausted T cells present by definition a lower ability to proliferate, investigating exhaustion in alloreactive T cells remains difficult today. Moreover, some of IRs are induced upon activation as a negative feedback loop, and our observations may be the result of a lower activation signal received by stimulated cells. Epigenetic marks of exhaustion, that could resist to stimulation^50^ could in the future help to a better understanding of allospecific T cell exhaustion after transplantation.

In conclusion, our study reveals that after kidney transplantation, a donor-specific T cell hyporesponsiveness develops after transplantation. The dysregulation of the CD226/TIGIT axis, with the increase of TIGIT over time leads to a decrease of CD4 + T cell response. Moreover, CD8 + PD1 + TIGIT + exhausted T cells increase with time, and participate in the decrease of T cell response after kidney transplantation. Future studies that include these subsets as biomarkers to monitor clinical endpoints or over- or underimmunosuppression are needed.

## Supplementary Information

Supplementary Information.

## Data Availability

The datasets used and/or analysed during the current study available from the corresponding author on reasonable request.

## Abbreviations

Tex: T cell exhaustion
HBV: Hepatitis B virus
HCV: Hepatitis C virus
CMV: Cytomegalovirus
PBMCs: Peripheral blood mononuclear cells
KTR: Kidney transplant recipients
DSA: Donor-specific antibodies
IR: Inhibitory receptors
MLR: Mixed lymphocyte reaction
