# Supplementary material for: The CD226/TIGIT axis is involved in T cell hypo-responsiveness appearance in long-term kidney transplant recipients
Source: Sci Rep. 2022 Jul 12;12:11821. doi: 10.1038/s41598-022-15705-6 (PMC9276733; doi:10.1038/s41598-022-15705-6)
Supplement: Supplementary file 1 — Supplementary Information. [file 41598_2022_15705_MOESM1_ESM.pdf]

Supporting documents

- Table of contents:

- Supporting document 1 (list of antibodies and supplies used in the study)
- Supporting document 2 (Phenotype analyses of exhaustion markers after kidney transplantation)
- Supporting document 3 (Mixed lymphocyte reaction)

Supporting Document 1. List of antibodies and supplies used in the study.

Flow cytometry analysis:

The list of antibodies included: CD4-BV510 (BD- Biosciences), CD8-BV786/785A (BD Biosciences), CD3-APC-H7/APC Cy7 (BD Biosciences), CD45RA -FITC/Alexa Fluor 488 (BD Biosciences), TIGIT - BB700/PerCP/Cy5.5 (BD Biosciences), CD197-PE-Cy7 (BD Biosciences), CD244-PE-CF594 (Thermofisher), intra-cellular Eomes-PE (BD Biosciences), CD160-Alexa Fluor647 (BD Biosciences), CD226- BV711 (BD Biosciences), CD279- BV650 (Thermofisher), CD366-BV421 (BioLegend), CD183- BB700/ PerCP/Cy5.5 (BD Biosciences), CD196- PE-CF594 (BD Biosciences), ICOS-PE (BD Biosciences), CXCR5- Alexa Fluor 647 (BD Biosciences), CD194- BD Horizon/ BV421 (BD Biosciences), HLA-DR- PECF594 (BD Biosciences), CD28-PE (BD Biosciences), CD57- PerCP/Cy5.5 (BD Biosciences), KLRG1-APC (BD Biosciences), CD25-PE-CF594 (BD Biosciences), intracellular FoxP3-PE (BD Biosciences), intracellular Helios- Alexa Fluor647 (BD Biosciences), CD127-BV421 (BD Biosciences), intracellular CTLA4- PE-Cy7 (BD Biosciences), CD161- BB700/PerCP/Cy5.5 (BD Biosciences), intracellular Granzyme B-Alexa Fluor647 (BD Biosciences), intracellular Perforin (BD Biosciences), Tim3- PE (BioLegend), CD56-BV650 (BD Biosciences).

intracellular cytokines:

PBMC were stimulated for 4 hours at 37°C with phorbol myristate acetate (PMA; 100 ng/ml, Sigma-Aldrich) and ionomycin (100 ng/ml, Calbiochem, Millipore) in the presence of GolgiStop (BD Biosciences). Fixation and permeabilization were performed using Cell Fixation & Cell Permeabilization Kit (Thermofisher), followed by staining with the appropriate antibodies, including FITC-Anti-TNF-a (Biolegend), BV510-anti-IL-2 (BD Biosciences), PE-Cy7-anti-IFN-g (clone B27; BD Biosciences).

Supporting Figure 2A

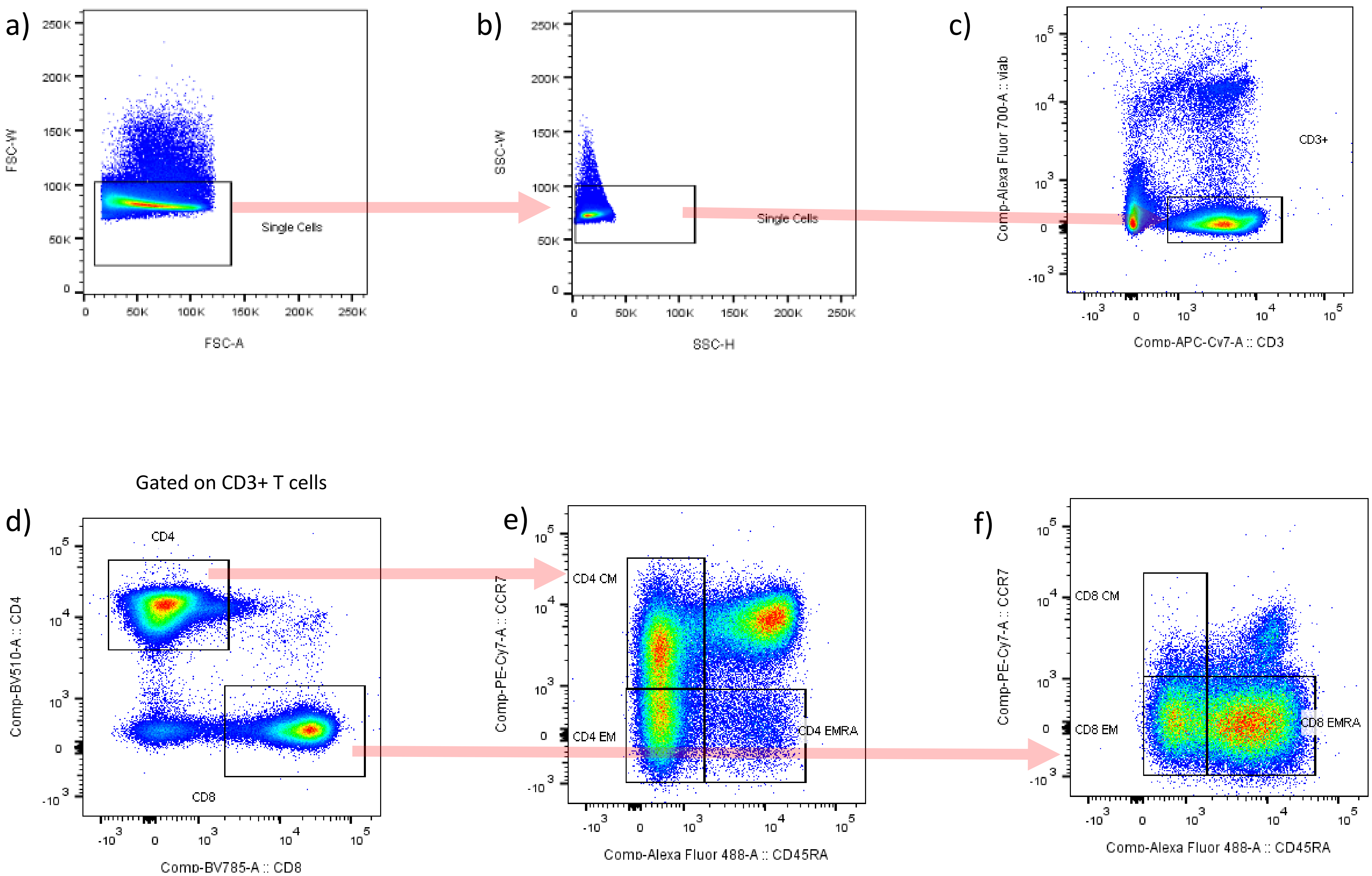

g) Inhibitory receptors and CD226 expression: gated on non-naive CD4+ T cells

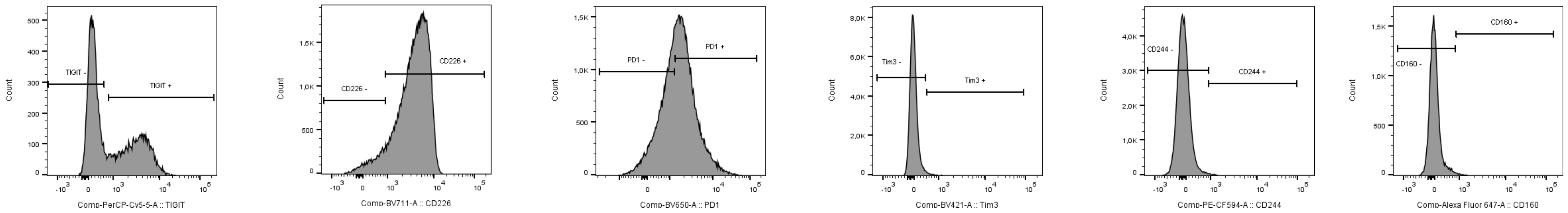

h) Inhibitory receptors and CD226 expression: gated on non-naive CD8+ T cells

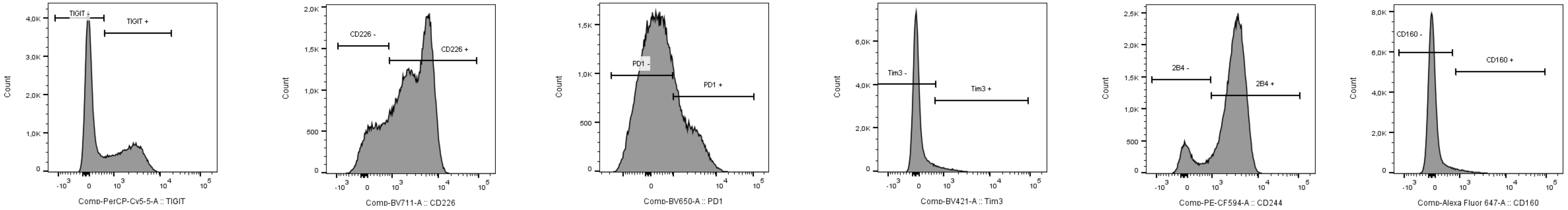

i) Gated on non-naive CD4+ T cells

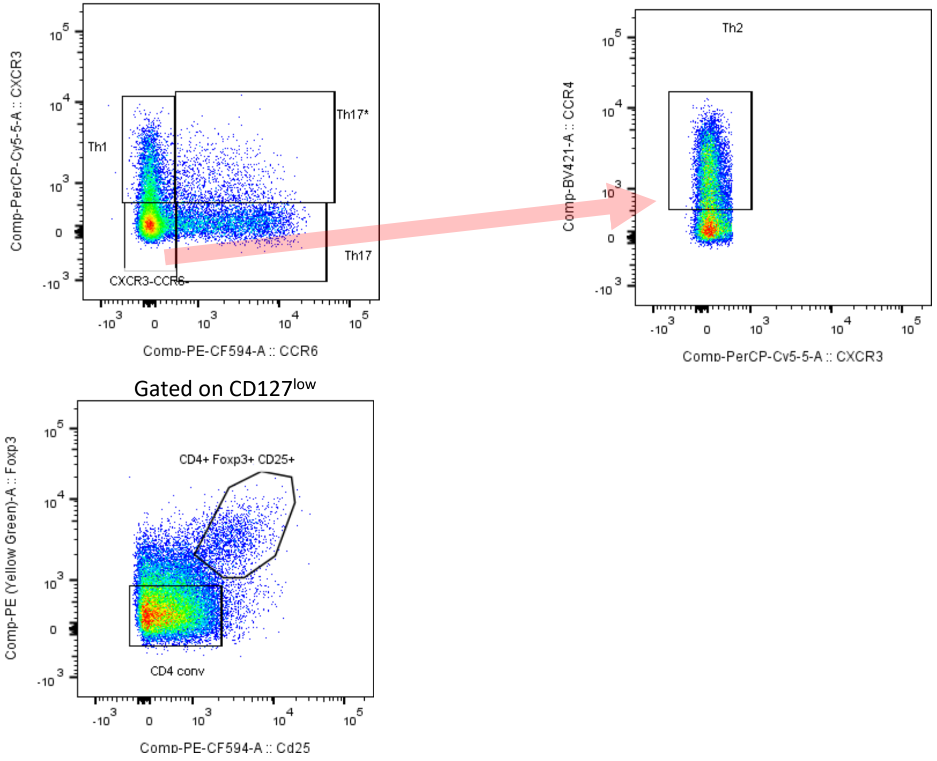

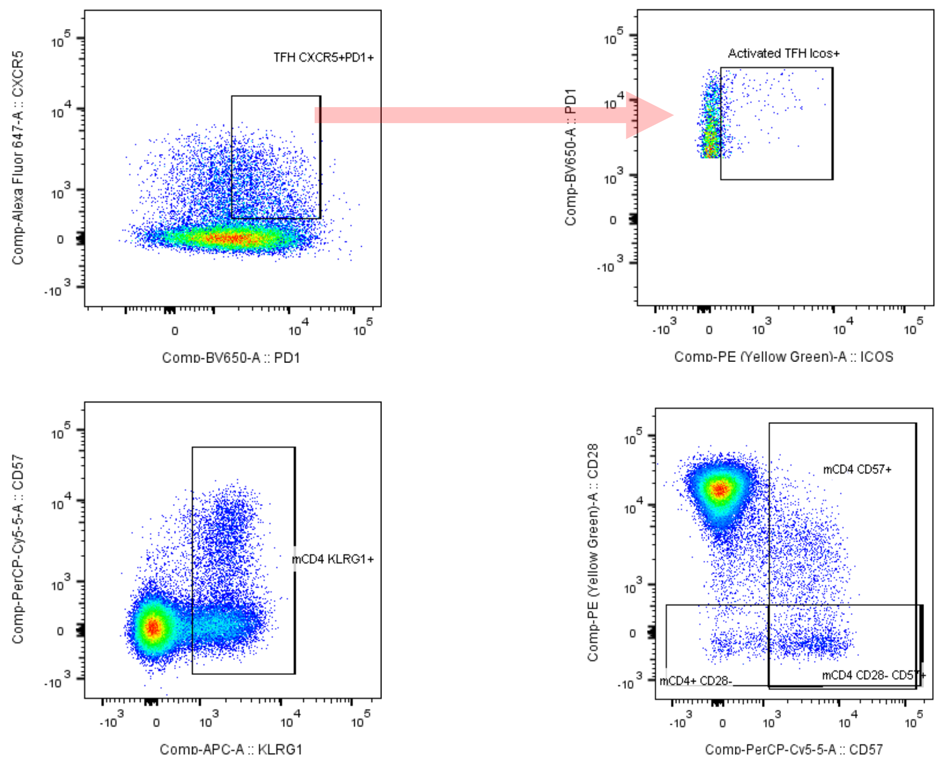

j) Gated on non-naive CD8+ T cells

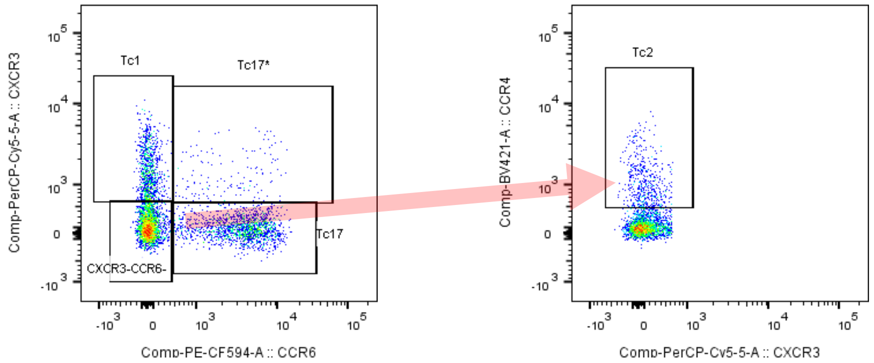

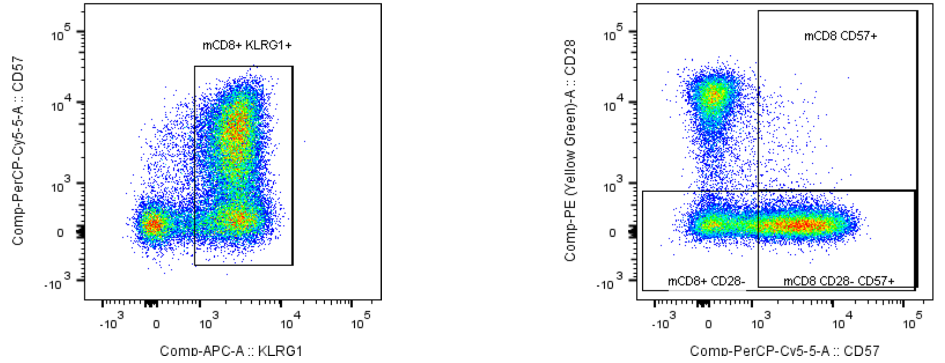

k) Functional analyses, gated on non-naive CD4+ T cells

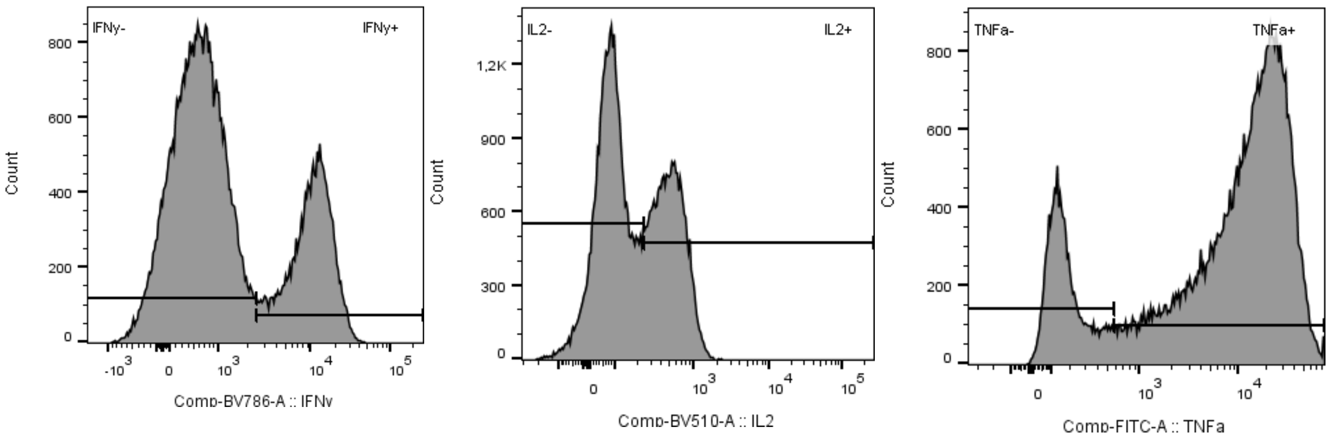

l) Functional analyses, gated on non-naive CD4+ T cells

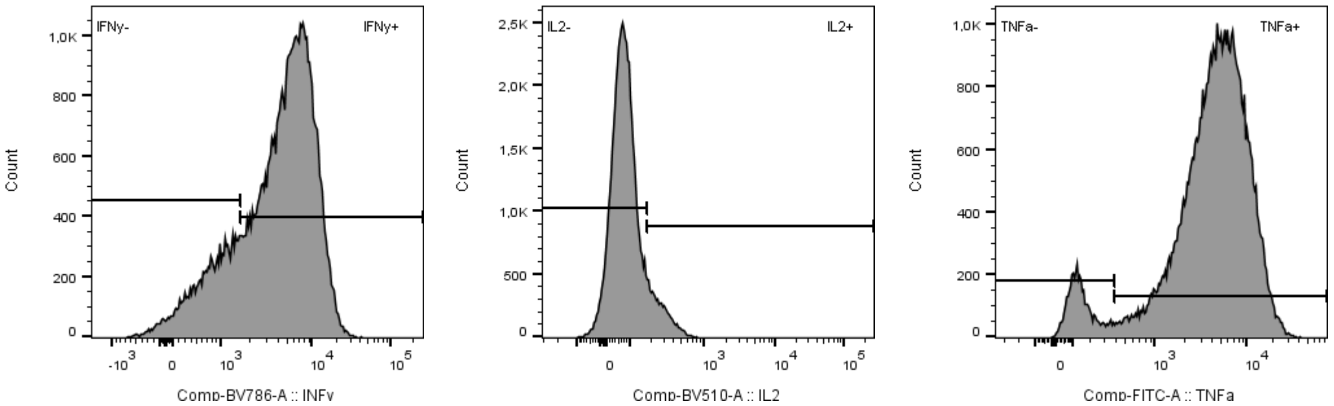

Supporting Figure 2B

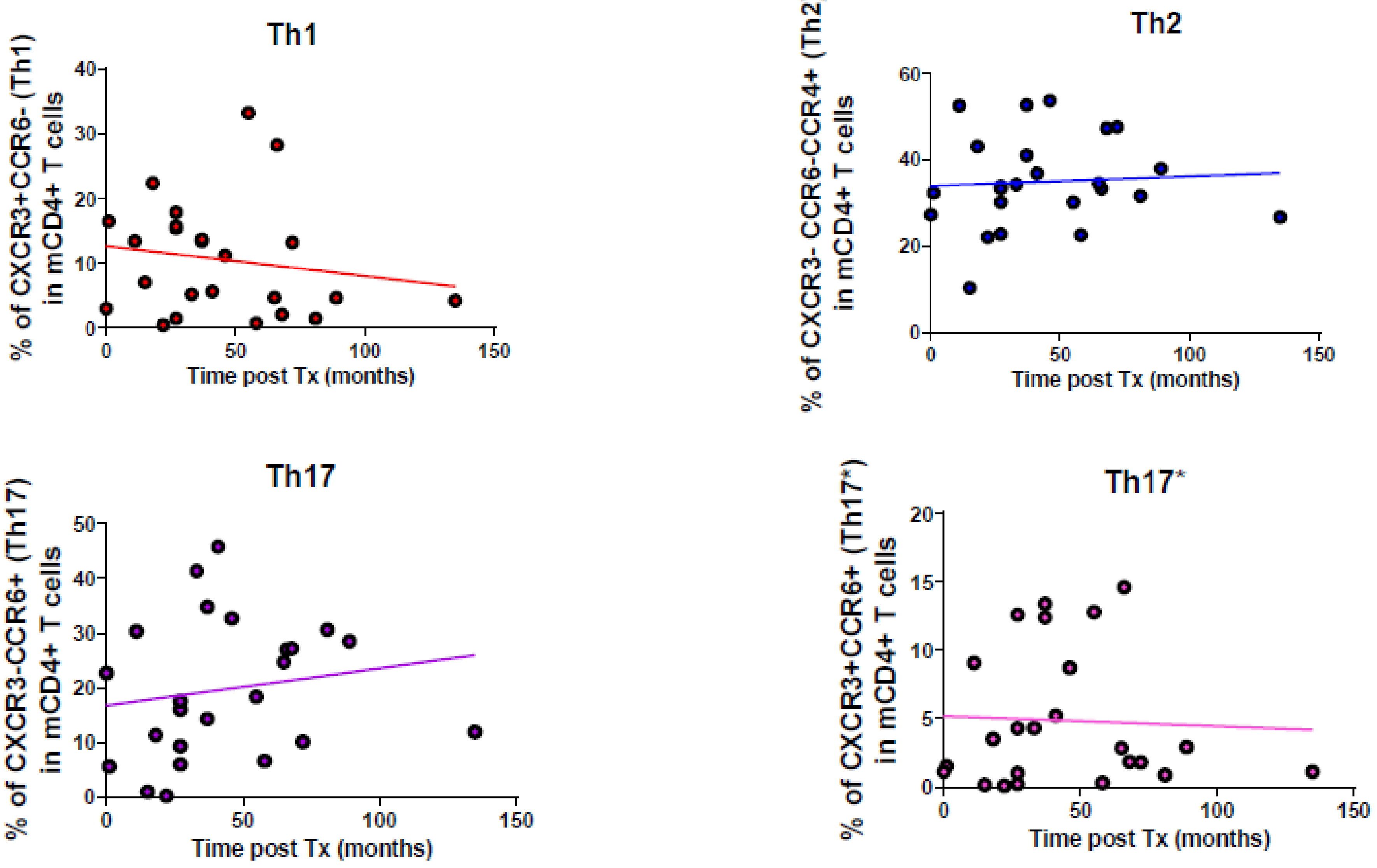

Supporting Figure 2C

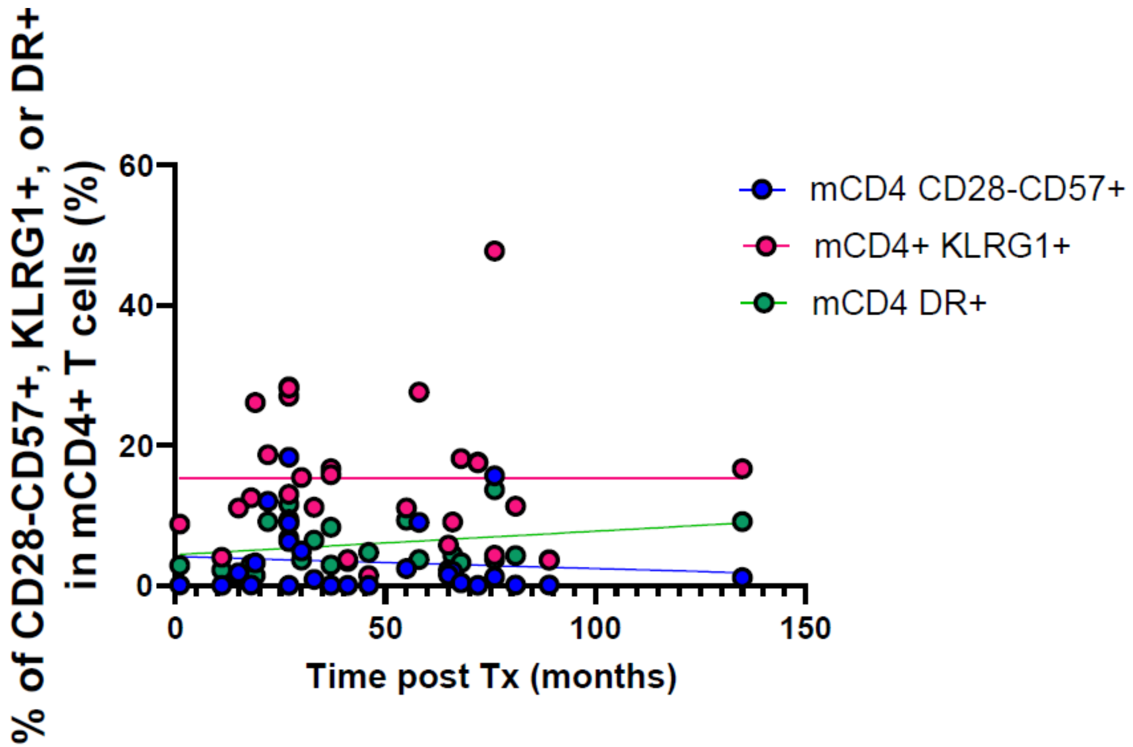

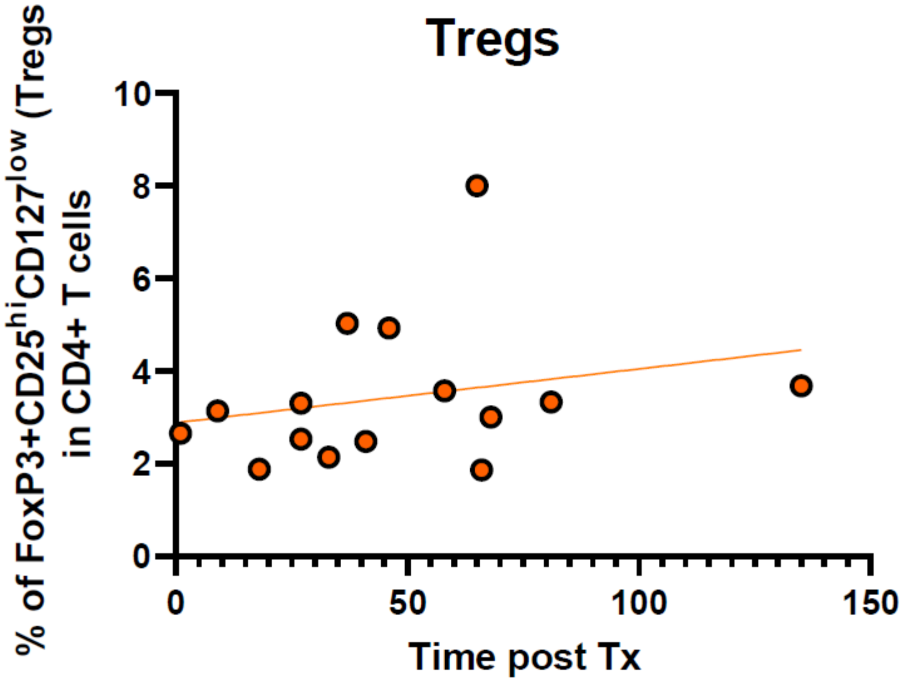

Supporting Figure 2D

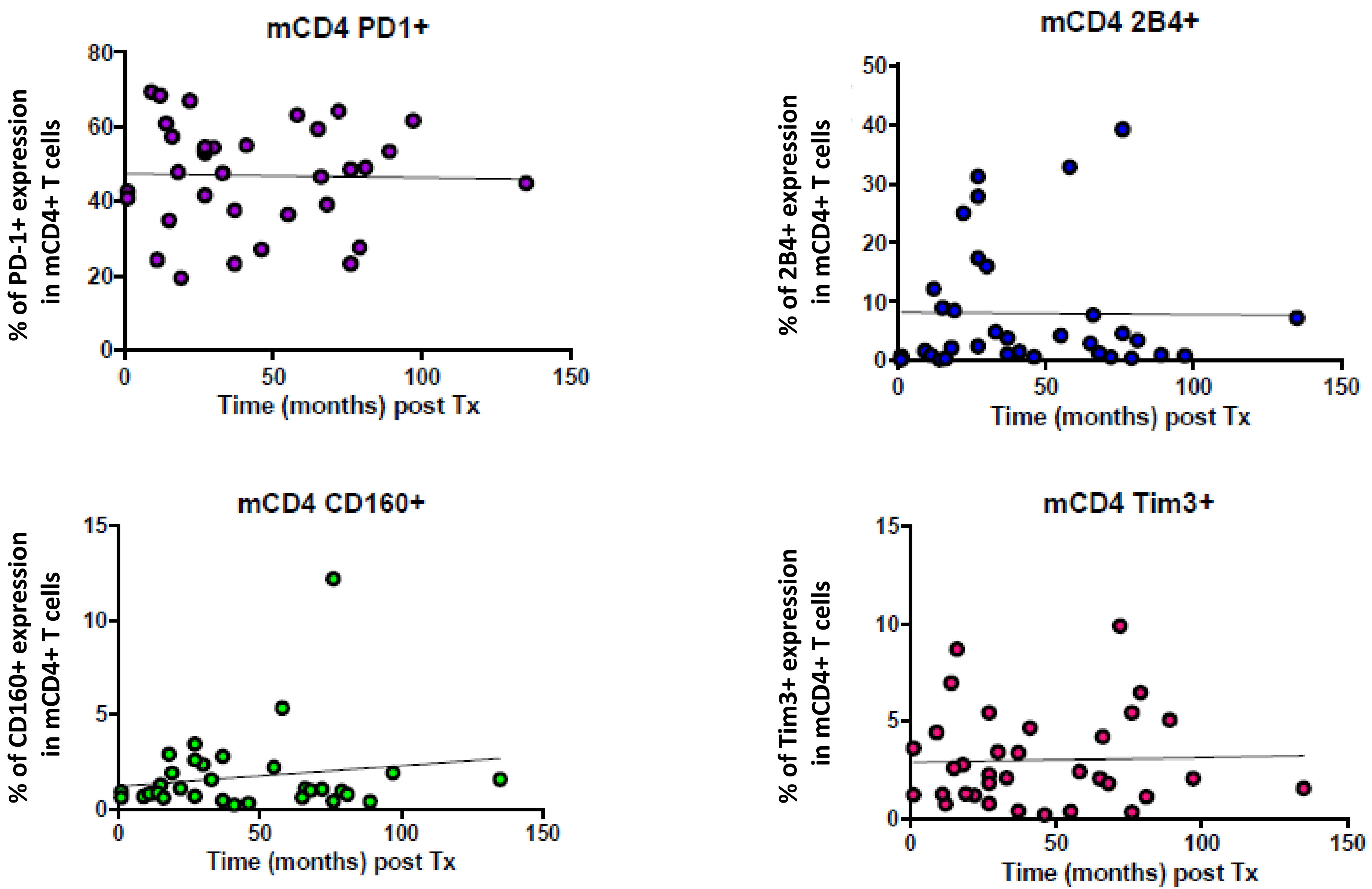

Supporting Figure 2E

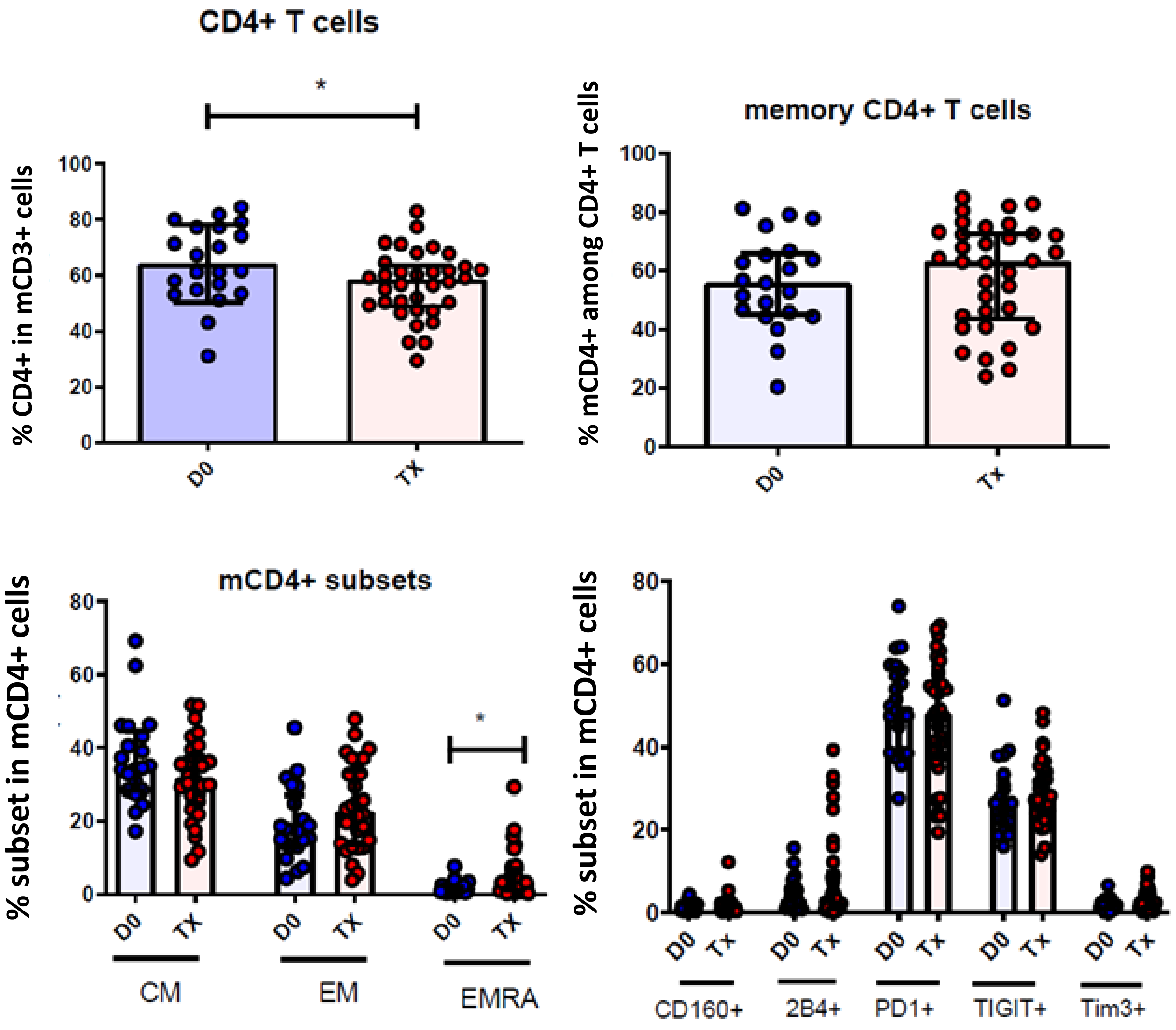

Variable	Dialysis patients (n=21)	Post- transplant recipients (n=30)	Results
Recipient age, year (IQR 25-75)	57 ± 17	52 ± 14	0.10
Recipient gender, male (%)	15 (71)	18 (60)	0.55
Initial kidney disease			0.42
- Glomerular	9	14	
- ADPKD/genetic	5	11	
- vascular	3	1	
- Unknown	4	4	
Positive CMV serology, yes (%)	13 (62)	16 (53)	0.58
Positive EBV serology, yes (%)	19 (90)	30 (100)	0.16

Table S2. Comparison between dialysis patients and kidney transplant recipients.

Abbreviations: ADPKD, Autosomal Dominant Polycystic Kidney Disease; CMV, Cytomegalovirus, EBV, Ebstein-Barr Virus.

Supporting Figure 2F

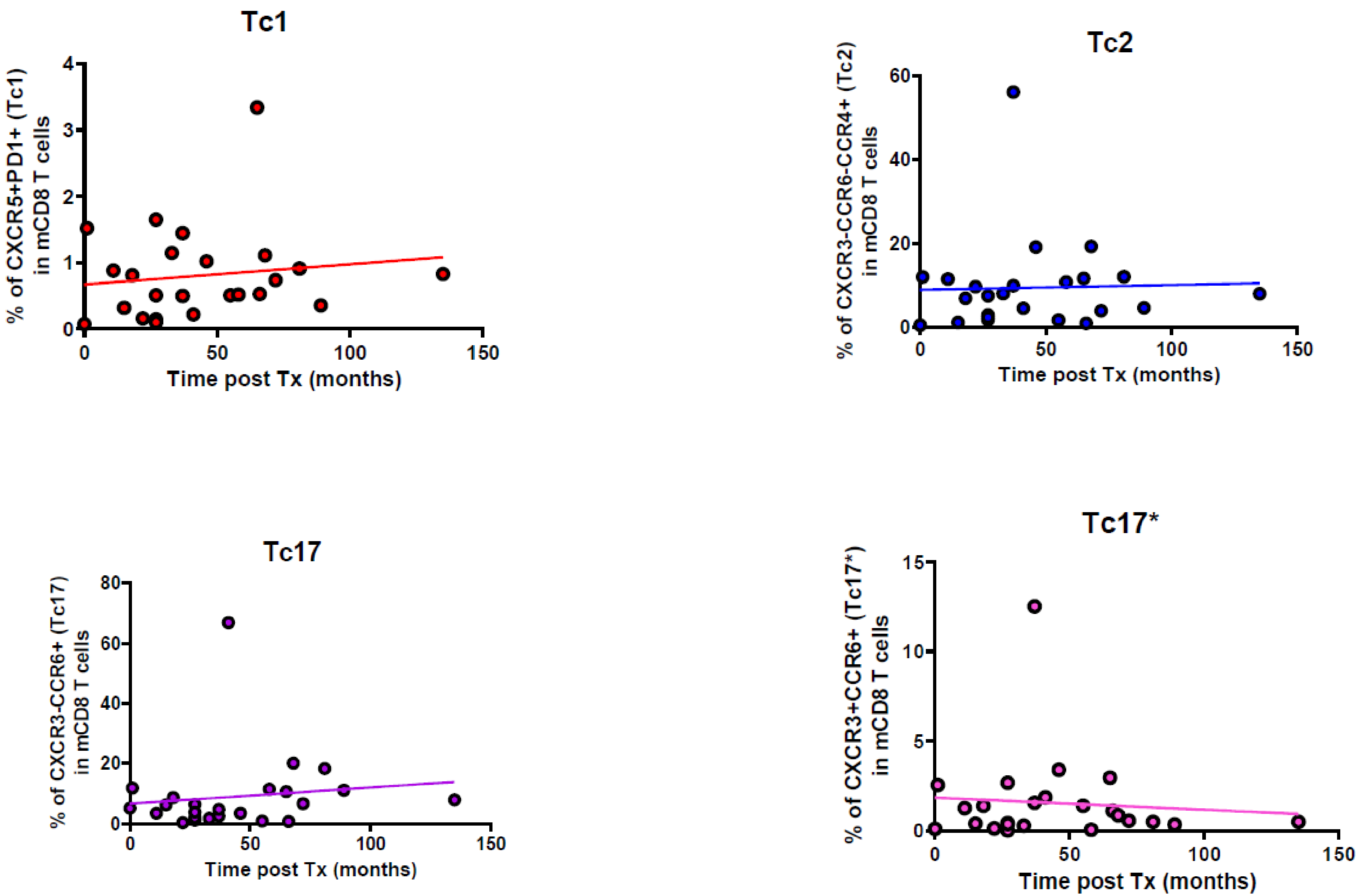

Supporting Figure 2G

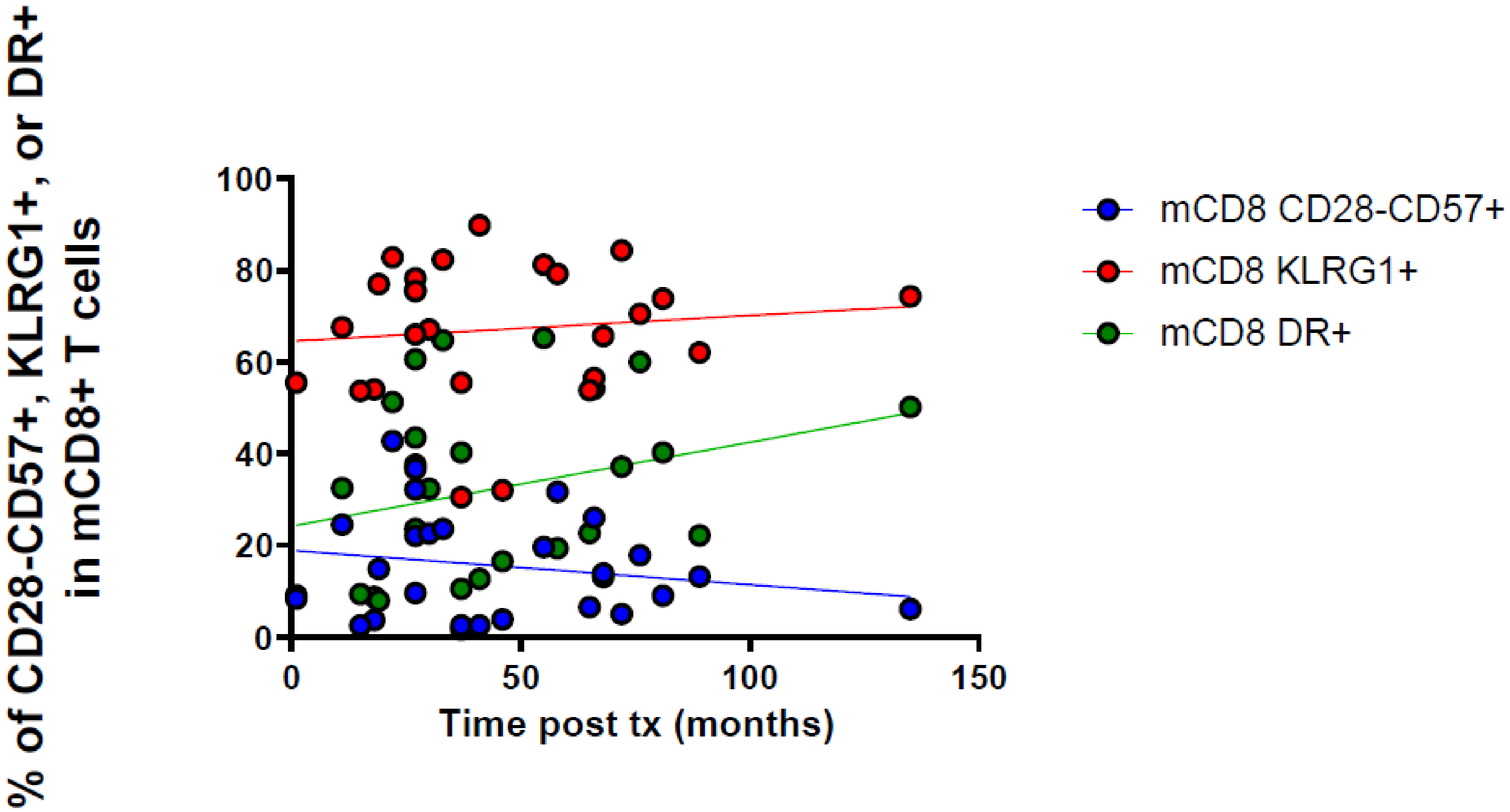

Supporting Figure 2H

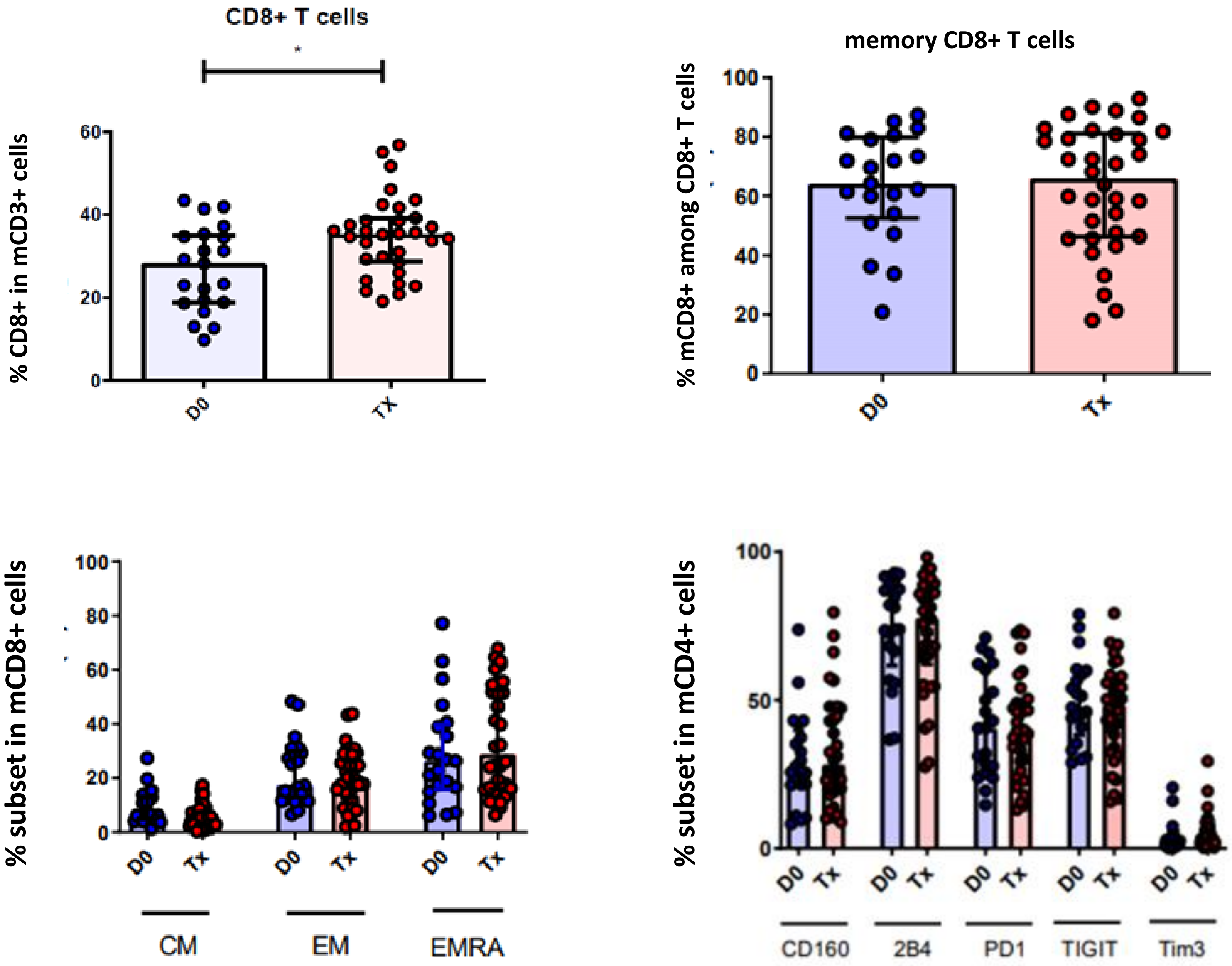

(A) Gating strategy

a & b) Identification of singlets using Forward Scatter (FSC)-W versus FSC-H (a) and Side Scatter (SSC) W vs SSC-H (b). Viable T cells were selected by gating on CD3+ cells and negative expression of the fixable viability strain (c). Within the lymphocyte population, CD4+ and CD8+ T cells were differentiated based on the surface expression of CD3+ and either CD4+ or CD8+ (d). Memory CD4+ (e) or CD8+ (f) T cells subsets were differentiated using the expression of CD45RA and CCR7 expression. Expression of the different inhibitory receptors, KLRG1, CD28, CD57, and TNFα, IFNγ, IL2 was analyzed in non-naïve CD4+ (g, k) and CD8+ T cells (h, l). The subsets were differentiated based on CXCR3, CCR4, CCR6, CCR5 expression. Tregs were defined using CD25, CD127, and FoxP3 expression.

(B) CXCR3+CCR6- (Th1), CXCR3-CCR6-CCR4+ (Th2), CXCR3-CCR6+ (Th17), and CXCR3+CCR6+ (Th17*) subsets in mCD4+ T cells with time post transplantation (Due to blood sample availability, only 24 patients were tested)

(C) CD28-CD57+, KLRG1+, DR+ (only 25 patients were tested, due to blood sample availability), and FoxP3+CD25^{hi}CD127^{low} (regulatory T cells) (n=15, due to blood sample availability) subsets in mCD4+ T cells with time post transplantation.

(D) Expression of each Inhibitory receptors in mCD4+ T cells with time post transplantation.

(E) Comparison of CD4+ naïve and memory T cell expression, in dialysis (D0) and transplant recipients.

(F) CXCR3+CCR6- (Tc1), CXCR3-CCR6-CCR4+ (Tc2), CXCR3-CCR6+ (Tc17), and CXCR3+CCR6+ (Tc17*) subsets in mCD8+ T cells with time post transplantation (only 25 patients were tested, due to blood sample availability).

(G) CD28-CD57+, KLRG1+, DR+ subsets in mCD8+ T cells with time post transplantation (only 25 patients were tested, due to blood sample availability)

(H) Comparison of CD8+ naïve and memory T cell expression, in dialysis (D0) and transplant recipients.

Supporting Document 3A

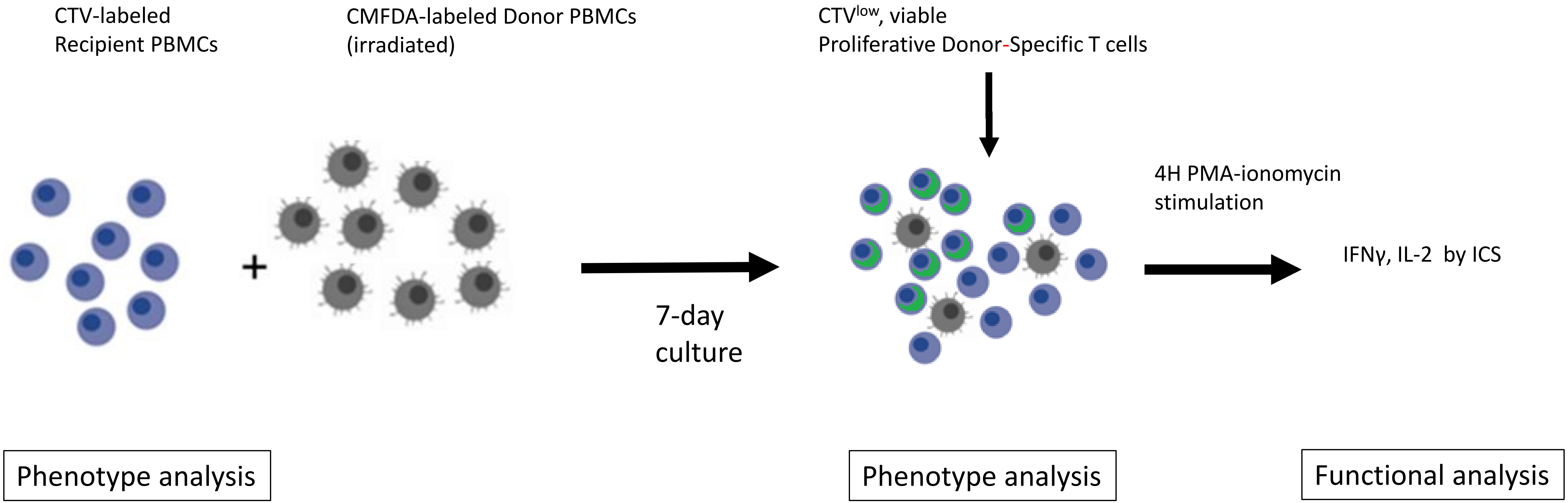

Supporting document 3

B Gating Strategy

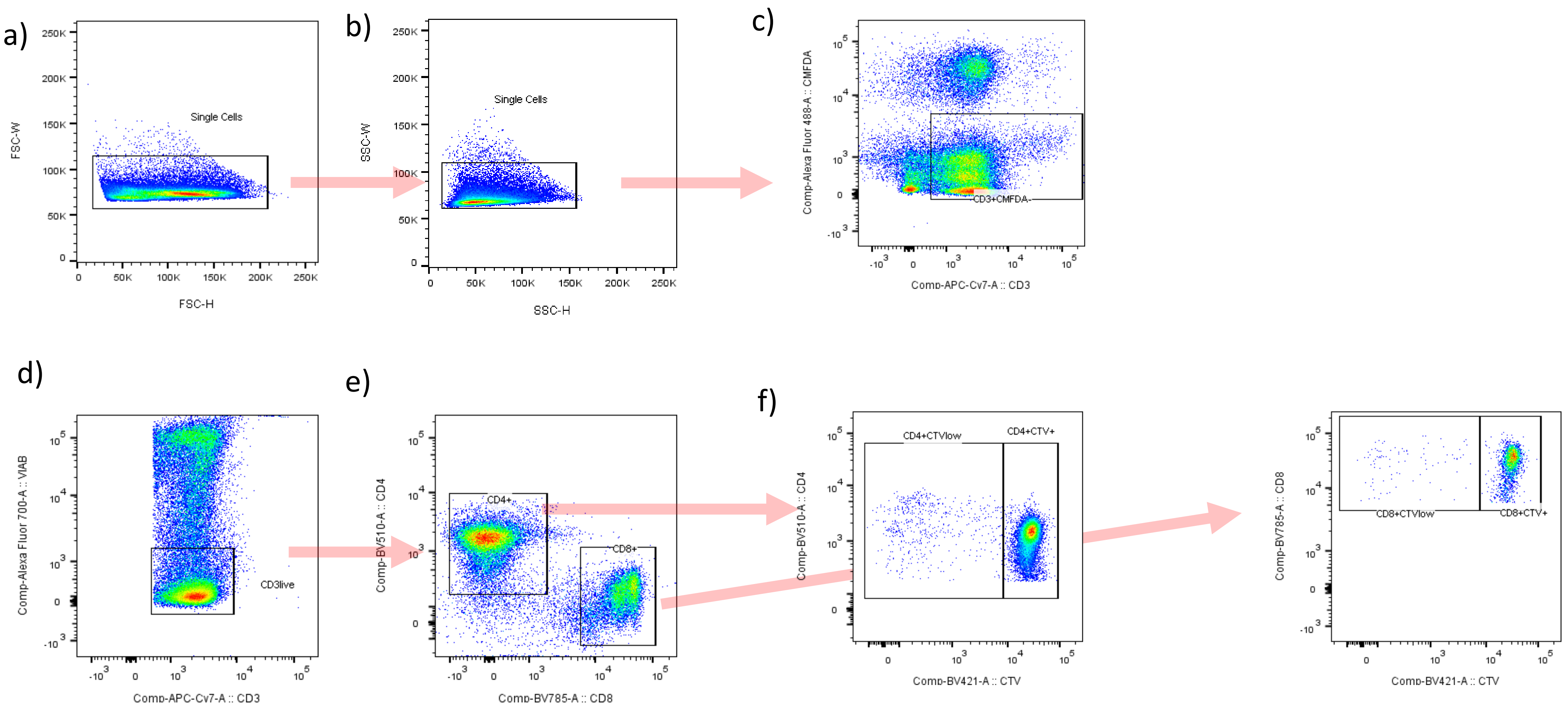

g) Inhibitory receptors and CD226 expression: gated on non-naive CD4+ T cells

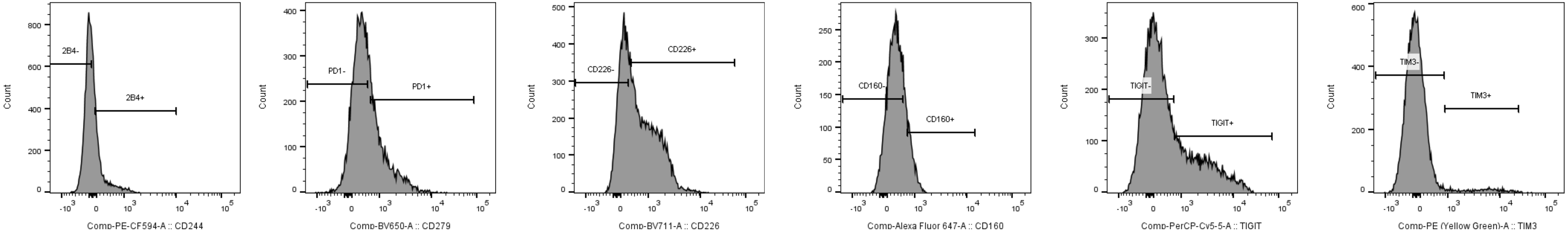

h) Inhibitory receptors and CD226 expression: gated on non-naive CD8+ T cells

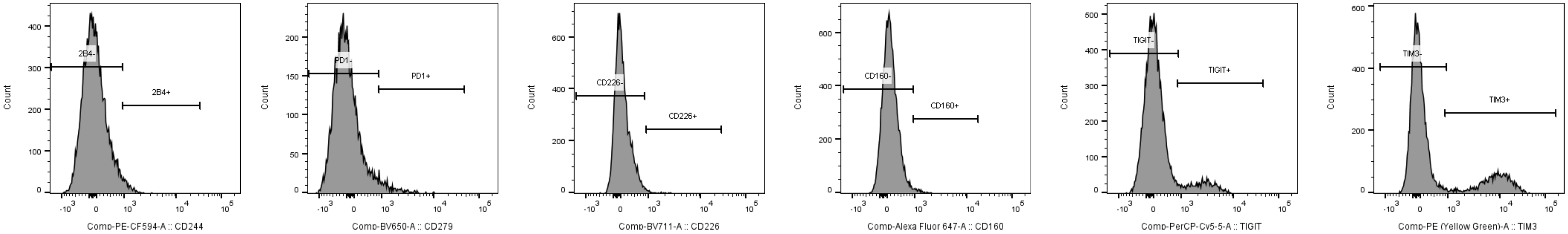

i) Functional analyses, gated on non-naive CD4+ T cells

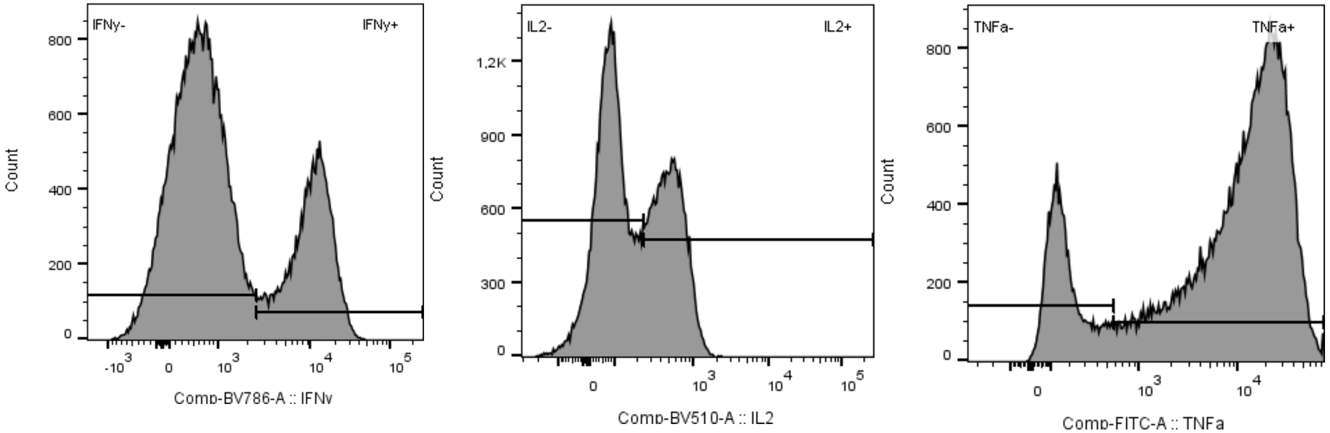

j) Functional analyses, gated on non-naive CD4+ T cells

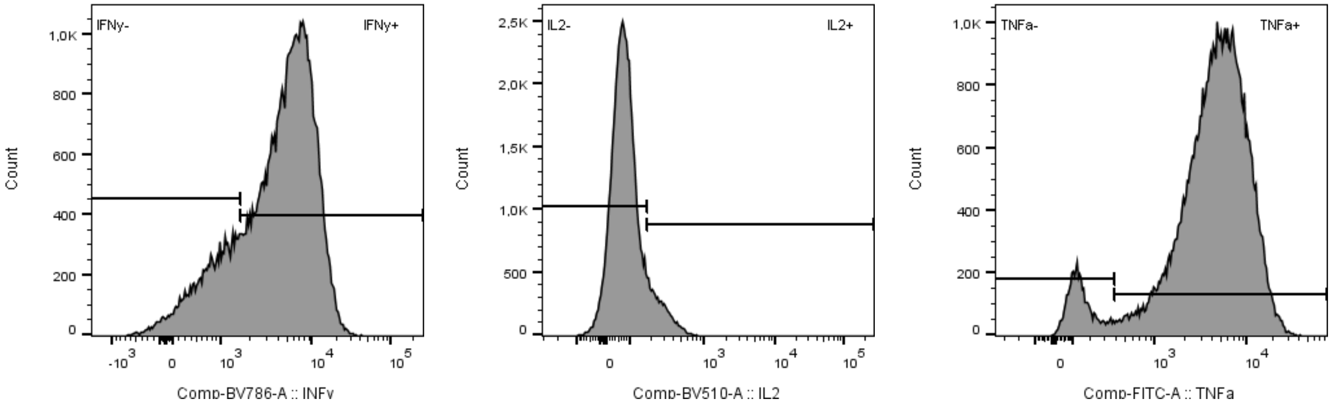

Supporting Figure 3C

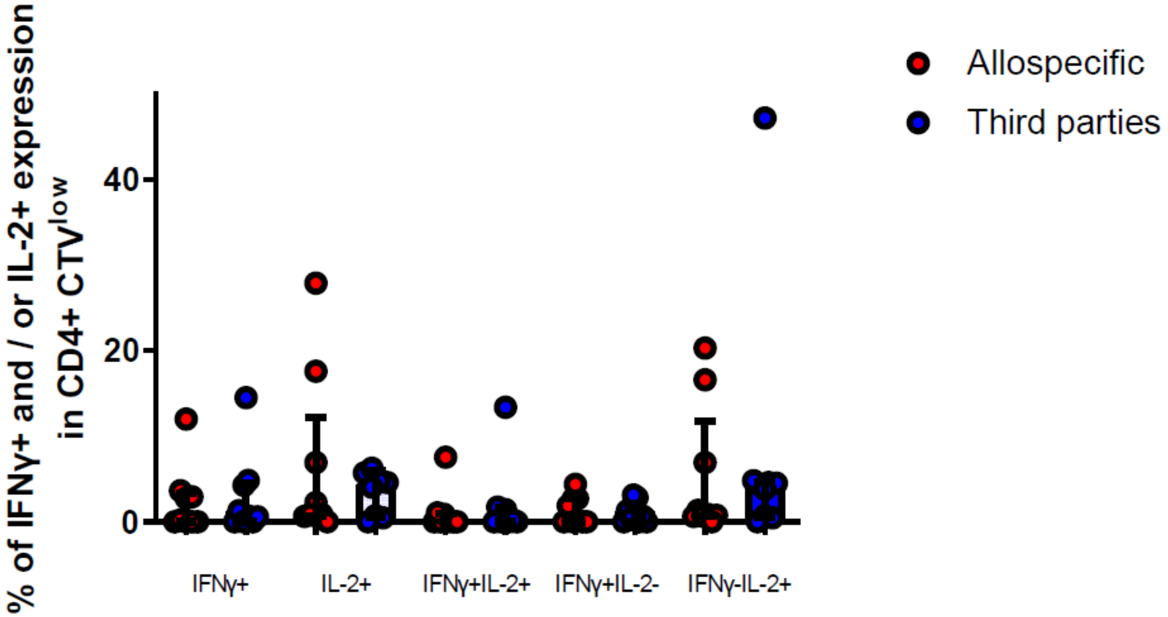

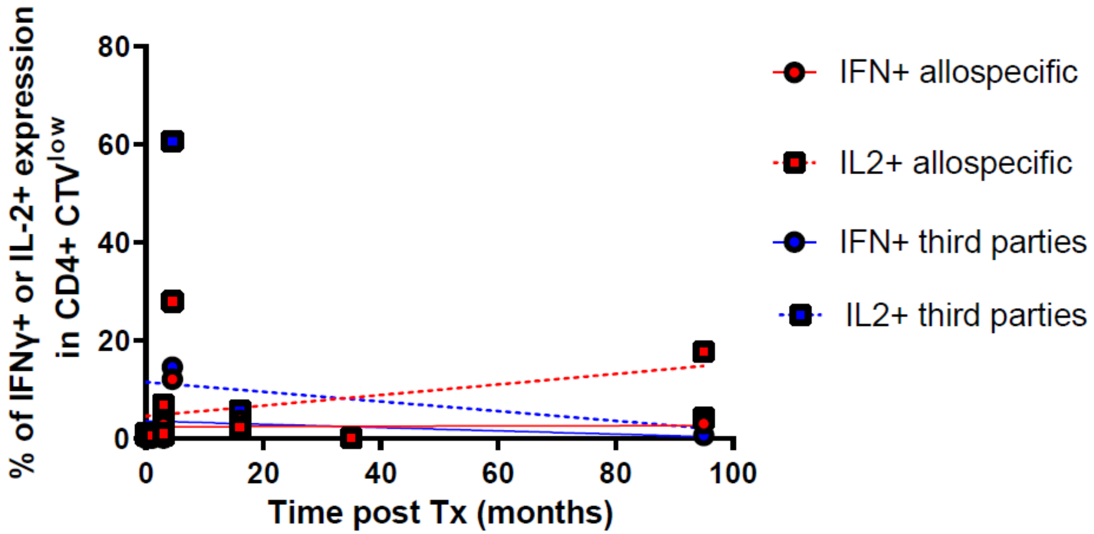

Supporting Figure 3D

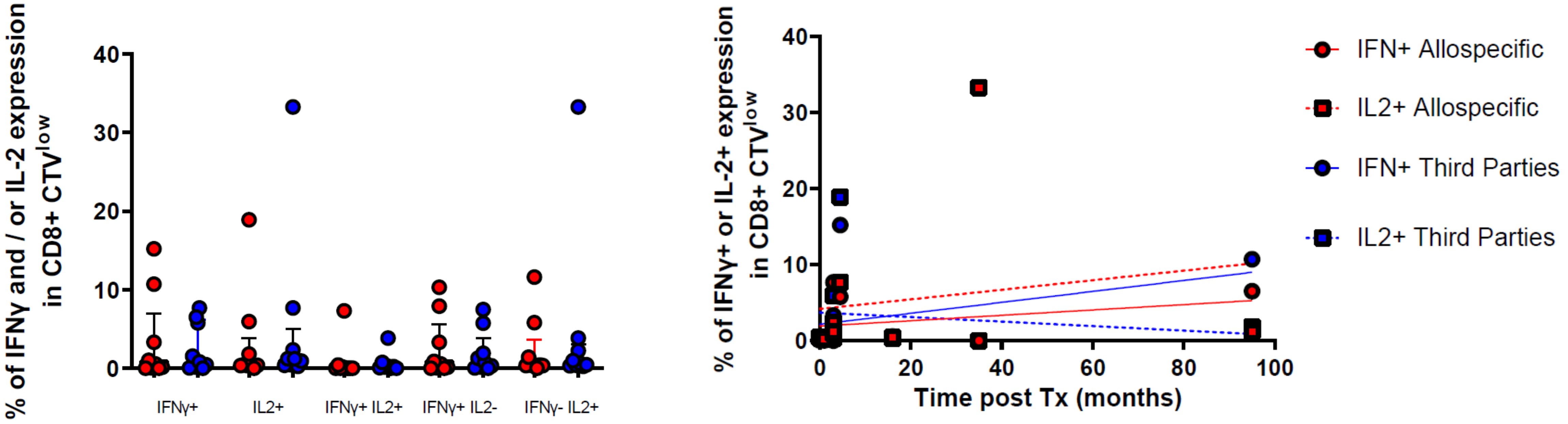

Supporting Figure 3E

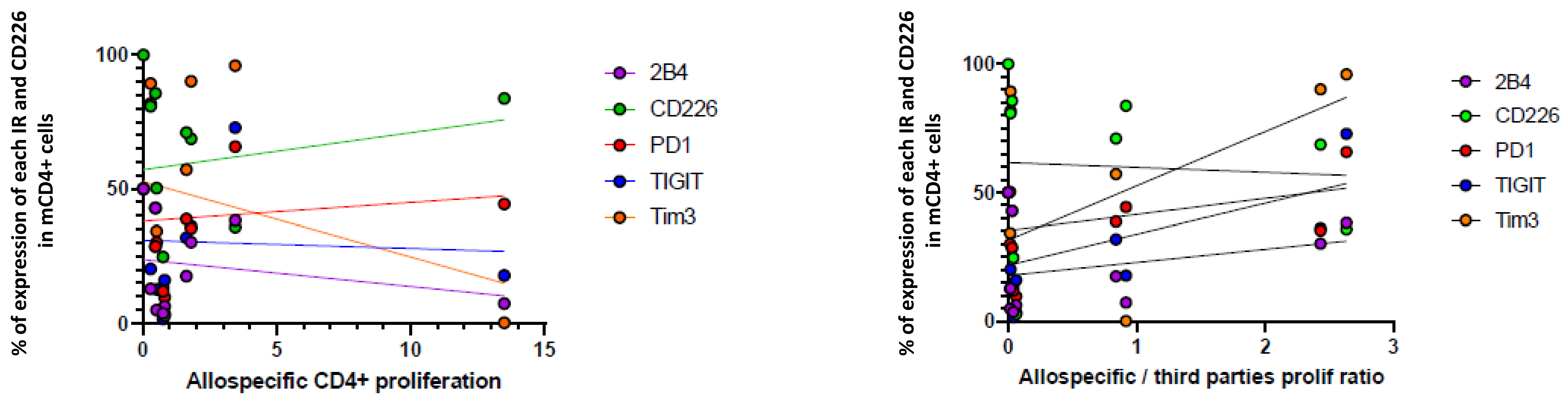

Supporting Figure 3F

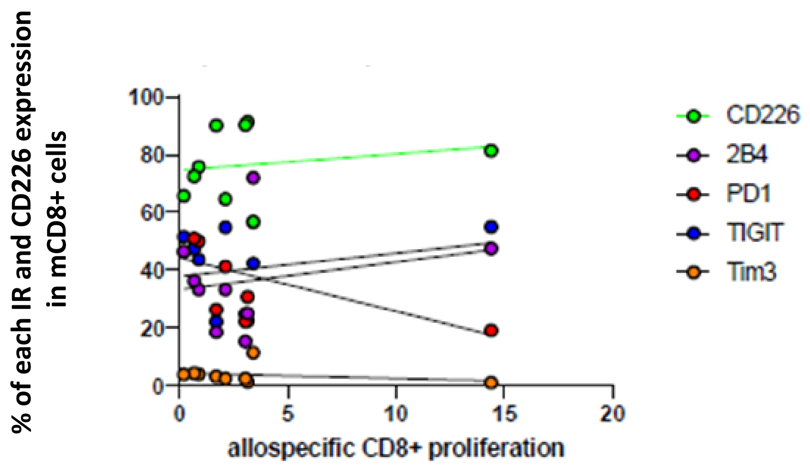

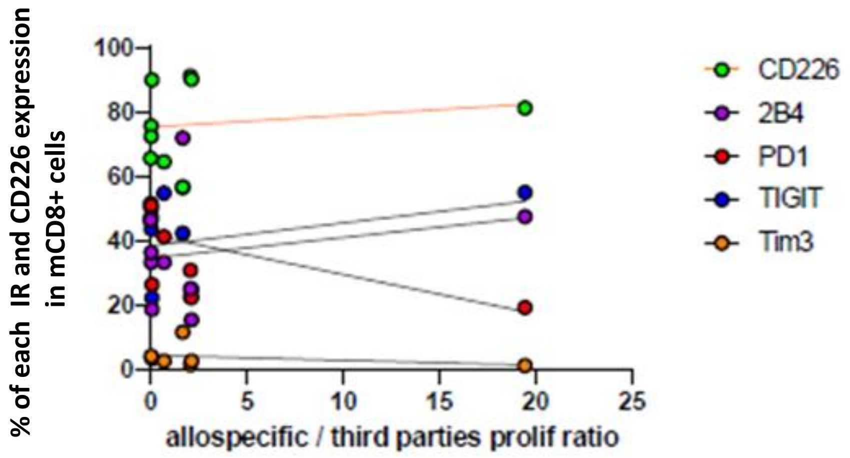

Supporting document 3. Mixed lymphocyte reaction

(A) Mixed Lymphocyte Reaction protocol

MLR assays were performed with CTV-labelled recipient cells, and CMFDA-labeled and irradiated stimulator cells, under four conditions: autologous (recipient cells used as stimulator cells), allo-specific (living kidney donor used as stimulator cells), third parties (from 4 different healthy blood donors equally distributed used as stimulator cells) and positive control with mitogenic agents (anti-CD2/CD3/CD28).

Phenotype analyses were performed before and after 7 days of culture.

After the culture, a functional analysis by Intra-cellular staining (ICS) of IL2, and IFNγ expression was performed after 4 hours of stimulation with PMA-ionomycin.

(B) Gating strategy

a & b) Identification of singlets using Forward Scatter (FSC)-W versus FSC-H (a) and Side Scatter (SSC) W vs. SSC-H (b). Responders T cells were selected by gating on CMFDA- cells (c), and the next negative expression of the fixable viability strain (d). Within the lymphocyte population, CD4+ and CD8+ T cells were differentiated based on the surface expression of CD3+ and either CD4+ or CD8+ (e). Proliferating cells were identified by gating on CTV- cells (f). Expression of the different inhibitory receptors, KLRG1, CD28, CD57, and TNFα, IFNγ, and IL2 was analyzed in non-naïve CD4+ (g, i) and CD8+ T cells (h, j).

(C) TNFα, IFNγ, and IL-2 expression in CTV^{low} CD4+ T cells after mitogenic restimulation (left), and correlation between cytokine expression and time post transplantation.

(D) TNFα, IFNγ, and IL-2 expression in CTV^{low} CD8+ T cells after mitogenic restimulation (left), and correlation between cytokine expression and time post transplantation.

(E) Correlation between expression of each IR expression in CD4+ T cells before stimulation and donor -specific (left) and allo-specific/ third-parties proliferation ratio (right).

(F) Correlation between expression of each IR expression in CD8+ T cells before stimulation and donor -specific (left) and allo-specific/ third-parties proliferation ratio (right).
